# Advances in Shape Memory Polymer Porous Materials: Fabrications, Microstructures, and Applications

**DOI:** 10.34133/research.0989

**Published:** 2025-12-08

**Authors:** Likai Hu, Fenghua Zhang, Lan Luo, Jinsong Leng

**Affiliations:** ^1^ Centre for Composite Materials and Structures, Harbin Institute of Technology (HIT), Harbin 150080, People’s Republic of China.; ^2^ Suzhou Research Institute, Harbin Institute of Technology (HIT), Suzhou 215100, People’s Republic of China.; ^3^ Guangzhou Institute of Future Additive Manufacturing, Guangzhou 510360, People’s Republic of China.

## Abstract

As an essential branch of smart materials, shape memory polymer materials (SMPs) have made substantial advances in fabrication strategies, microstructure design, and response methods. Shape memory polymer porous materials (SMPPMs), combining the advantages of SMPs and porous materials, feature lightweight properties, tunable micro/nanostructures, large specific surface areas, and programmable shapes, which have attracted important attention across a wide range of applications. This study focuses on 2 types of SMPPMs: shape memory foams and shape memory aerogels. This review systematically examines the fabrication strategies for SMPPMs, including gas foaming, template, freeze-drying, and 4-dimensional printing methods, deeply analyzes the impact of fabrication strategies on their micro/nanostructures, and summarizes their latest applications in areas such as smart thermal protection systems for aerospace, minimally invasive biomedical devices, and high-sensitivity smart sensors. This review analyzes the current state of research and future trends in SMPPMs from multiple perspectives, including material design, structural design, and response strategies. The design of SMPPMs requires integration with actual application needs, achieved through appropriate selection of polymer matrices and optimized micro/nanostructure designs to improve material performance. Furthermore, the review also introduces current challenges and development trends related to SMPPMs, including advanced, sophisticated, large-scale preparation strategies, and efficient, rapid, precise driving methods, as well as applications that integrate multiple disciplines and fields.

## Introduction

With the advancement of science and technology, smart materials are comprehensively enhancing the quality of human life in an all-round way [1–5]. As a key category of intelligent materials, shape memory polymers (SMPs) have garnered significant research interest due to their unique stimulus-responsive behavior [6–11]. The unique shape memory behavior of SMPs originates from their intricate molecular architecture, in which the fixed phase and reversible phase function synergistically to produce the shape memory effect. The shape memory characteristics confer upon SMPs a range of advantageous features, including highly programmable deformation capabilities, multiple stimulus-responsive modes, and excellent processability, thereby establishing SMPs as a critical material for achieving integrated structural and functional performance. Owing to these attributes, SMPs demonstrate extensive application potential across diverse fields such as aerospace deployable structures, biomedical implants, smart textiles, and soft robotics [12–16]. While SMPs offer remarkable programmability and stimulus responsiveness, their applications in scenarios requiring extreme lightweight, high compressibility, or specific interfacial properties are often constrained by their typically solid, nonporous bulk morphology. Conversely, conventional porous materials are prized for their low density, high specific surface area, and tunable permeability. However, they generally lack active, stimulus-responsive controllability over their structure and properties once fabricated, limiting their functionality to passive roles.

Advances in micro/nanotechnology and the emergence of integrated design concepts for material structure and function have generated novel synergies between smart materials and porous structures, leading to the development of smart porous materials such as shape memory polymer porous materials (SMPPMs), including shape memory polymer foams (SMPFs) and shape memory polymer aerogels (SMPAs). The integration of shape memory functionality into porous matrices gives rise to SMPPMs, which effectively address the bottlenecks inherent to the individual material systems. SMPPMs synergize the dynamic, programmable shape recovery of SMPs with the structural advantages of porous materials, enabling active control over properties such as porosity, permeability, and compressibility in response to external stimuli. This convergence significantly expands the application horizon beyond what is achievable with either SMPs or conventional porous materials alone.

Since the initial reports in the 1990s, SMPPMs have transitioned from the investigation of standalone SMPFs to a more sophisticated paradigm characterized by the synergistic development of both SMPFs and SMPAs. The earliest SMPPMs were shape memory polyurethane (SMPU) foams developed by Mitsubishi Heavy Industries (MHI). These materials have since progressed toward commercialization and have been applied in diverse fields such as construction and healthcare. As application environments increasingly demanded enhanced thermal stability, medium-temperature SMPFs were introduced, including shape memory epoxy (SMEP) foams and shape memory polyvinyl alcohol (PVA) foams. Among these, SMEP foams have been successfully validated for aerospace applications. Compared to conventional foams, SMPFs exhibit superior specific modulus and specific strength, along with precisely controllable shape recovery capabilities. These materials can be compressed for compact storage and transportation, and they recover to their original volume upon exposure to external stimuli [17,18]. The availability of diverse stimulation methods further expands the application potential of SMPFs across multiple domains.

**Fig. 1.**
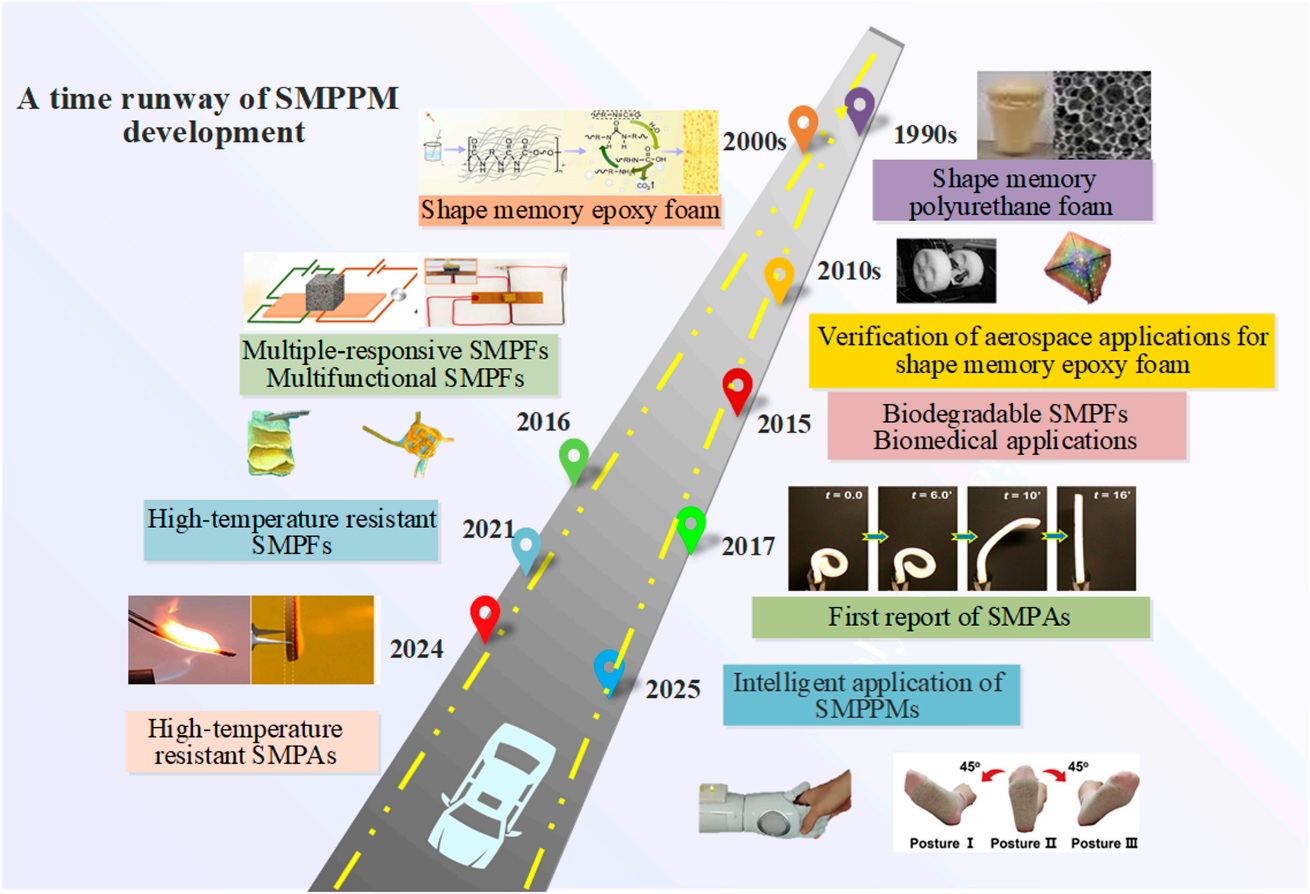
A time runway of SMPPM development.

SMPAs exhibit even higher porosity and lower density than SMPFs [19]. They are primarily fabricated via sol–gel or template-based approaches, yielding 3-dimensional (3D) nanoscale porous architectures with porosity typically exceeding 90%. This results in ultra-low densities ranging from 0.02 to 0.2 g/cm^3^ and high specific surface areas between 10 and 1,000 m^2^/g [20]. Research on SMPAs commenced in 2017, and, similar to SMPFs, the first generation of SMPAs was based on SMPU. The demand for intelligent thermal protection materials in aerospace thermal protection systems has accelerated the development of high-temperature SMPAs, such as shape memory polyimide (SMPI) aerogels and shape memory phenolic aerogels. Entering the 2020s, SMPPMs are evolving toward greater diversification, precision, and functional integration, as illustrated in Fig. 1. This paper systematically reviews the development of SMPPMs, encompassing their preparation methods, microstructures, stimulus-response modes, and primary applications. The overarching aim is to provide clarity and direction for future research in this field. These materials combine the stimulus-responsive characteristics of SMPs with the intrinsic advantages of porous structures, offering low weight, high compressibility, and tunable micro- and nanoscale architectures. Their versatility has enabled applications across a wide range of fields. For example, biocompatible SMPU foams are employed in the fabrication of aneurysm embolization devices, while thermally stable SMPAs are integrated into thermal insulation systems. Additionally, SMPPMs are being explored for use in apparel [1], soft robotics [21,22], and intelligent control systems [23].

**Fig. 2.**
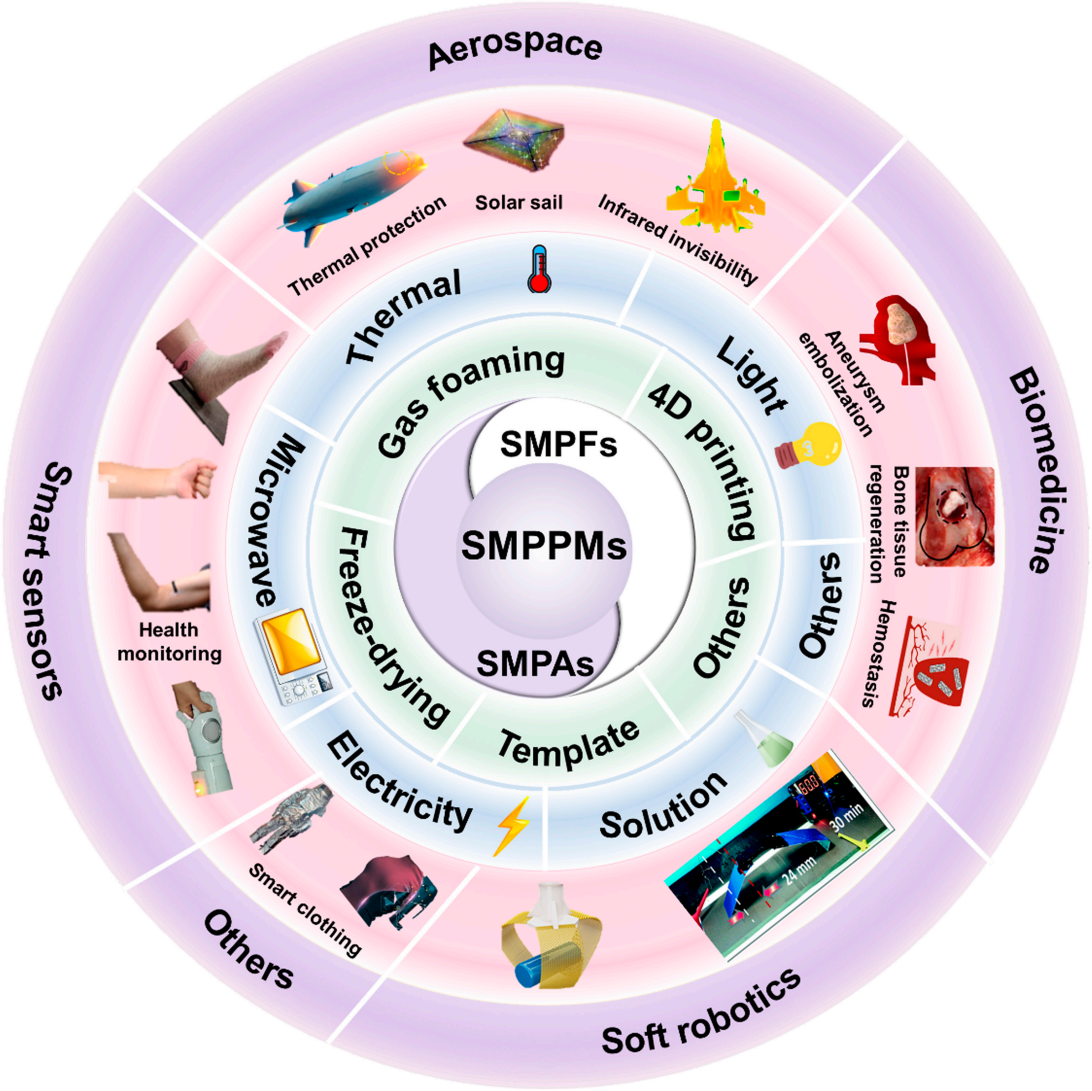
Preparation methods, actuation methods, and applications of intelligent porous materials.

Here, we summarize the latest significant advances of SMPPMs. The performance of SMPPMs is closely related to their microstructure, and their multifunctional properties are directly linked to their chemical structure. Therefore, recent research on SMPPMs has focused on the development of new materials and the study of novel preparation techniques. The paper reviews the material classification system and design principles, key preparation strategies and structural control methods, diverse stimulus-driven mechanisms and functional realization pathways, as well as cutting-edge application scenarios of SMPPMs in recent years (Fig. 2). It also analyzes the current challenges faced by smart porous materials. Finally, the paper looks ahead to the future development prospects and challenges of SMPPMs. We hope to gain a more systematic understanding of the development of SMPPMs.

## Fabrication Strategies

The fabrication process of SMPPMs and the selection of process parameters significantly influence the performance of the resulting materials. Accordingly, researchers typically select suitable formation techniques based on the intended application and performance requirements of the target product [24]. The pore structure of SMPPMs plays a crucial role in determining their mechanical properties, and this structure is directly influenced by the formation process [25,26]. Therefore, designing the pore architecture through appropriate fabrication strategies remains a key focus in current SMPPM research. Initially, SMPPMs were predominantly fabricated using conventional methods such as gas foaming, template-based techniques, and freeze-drying. However, as research into SMPPMs has advanced, innovative pore-forming approaches—such as microwave foaming, 4D printing, and laser foaming—have also emerged and continue to evolve.

### Gas foaming method

Gas foaming is a widely employed method for the preparation of SMPPMs [27,28], which operates via physical or chemical mechanisms. Physical foaming incorporates volatile agents into SMPs; heating evaporates them, entrapping gas within the polymer matrix to form pores. Chemical foaming relies on foaming agents decomposing into gases during curing. Certain polymers (e.g., phenolic resins) may also release volatile byproducts during controlled curing, facilitating agent-free foaming.

**Fig. 3.**
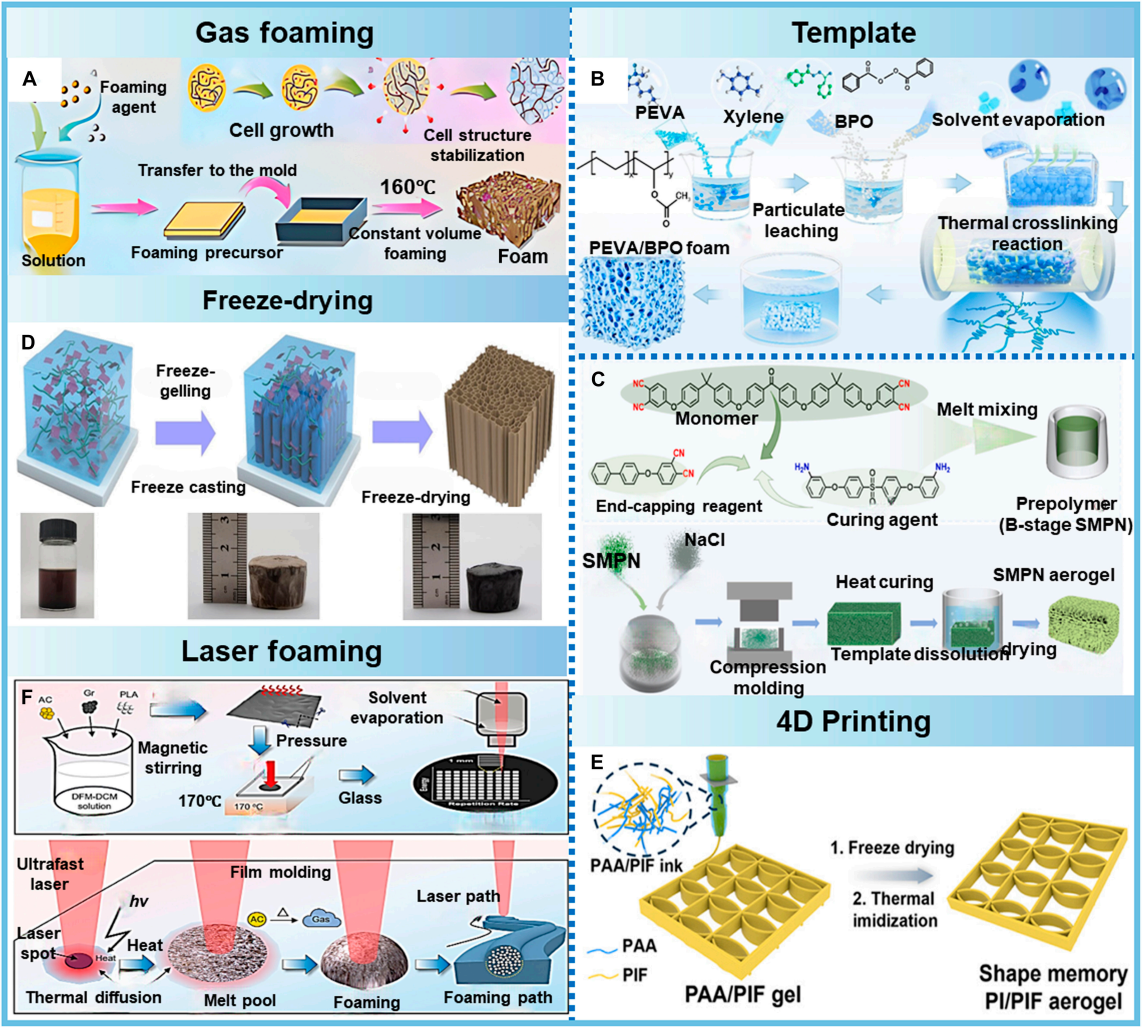
Preparation strategies of SMPFs. (A) Schematic of the gas foaming method. Reprinted with permission from [32]. (B and C) Schematic of the template method. Reprinted with permission from [33,36]. (D) Schematic of the freeze-drying method. Reprinted with permission from [41]. (E) Schematic of the preparation of SMPFs by 4D printing. Reprinted with permission from [48]. (F) Schematic of the laser foaming method. Reprinted with permission from [55].

SMPU foams predominantly utilize gas foaming. Early methods employed CO₂ from isocyanate reactions. Walter et al. [29] introduced water as a foaming agent, exploiting its reaction with isocyanate to generate gas. This produced SMPU foams with uniform pores (150 to 250 μm) and 88% porosity. Similarly, Ren et al. [30] used the isocyanate–water reaction, creating macroporous gradient structures. Reaction gas drove bubble aggregation/growth, while pore wall strength variations along the expansion direction enabled gradient formation. Tian et al. [31] employed 1,5-diaminopentane carbamate as a combined latent curing/foaming agent for SMPU foam fabrication. The primary advantage of gas foaming is achieving high porosity. Additionally, Zhou et al. [32] fabricated shape memory polyphosphazene foams using azodicarbonamide as a blowing agent, achieving integration of SMPFs with phase-change materials and endowing the material with flame-retardant and multi-drive properties (Fig. 3A). Significant drawbacks exist: Physical foaming suffers from imprecise control over solvent evaporation, often causing inhomogeneous structures. Additionally, many foaming agents contain toxic components, posing environmental hazards.

### Template method

The template method is a highly effective approach for fabricating porous materials by removing a predefined template to create pores. This technique enables precise control over the microstructure and pore dimensions of SMPPMs, allowing for tailored pore distributions and organized architectures to meet diverse functional requirements.

Numerous early studies employed the salt template method to fabricate SMPFs and demonstrated that the pore size could be controlled by adjusting the size of salt particles. Hui et al. [33] combined BPO (benzoyl peroxide)/NaCl particles, thermally crosslinking them to create shape memory PEVA [poly (ethylene-co-vinyl acetate)]/BPO composites; dissolving the template yielded SMPFs with pore size controllable via NaCl particle size (Fig. 3B). Chen et al. [34] co-cured salt particles mixed into a polymer matrix to produce shape memory EVA foam. Hu et al. [35] nanoscale-mixed a shape memory boron-containing phenolic prepolymer with NaCl, synthesizing shape memory phenolic aerogel. They leveraged phenolic curing/dehydration, enabling the salt template to induce secondary pore formation, resulting in simultaneous micropores and nanopores. Additionally, Hu et al. [36] employed NaCl as a template to fabricate SMPNAs (shape memory phthalonitrile aerogels), with the preparation process depicted in Fig. 3C. This series of studies collectively demonstrates the effectiveness and flexibility of salt templates in creating complex hierarchical pore structures. Beyond conventional salt templates, researchers have explored structural templates to create interconnected porous networks. For instance, Zhao et al. [37] employed nickel foam to fabricate shape memory composite foams, achieving high interconnectivity for liquid transport. He et al. [38] utilized 3D porous melamine foam (MF) as a template, vacuum-impregnating MF@MA (melamine foam modified by MXene/AgNW) elastic foam with polyethylene glycol to develop multifunctional integrated shape memory composite foams. These studies highlight the unique advantage of structural templates in conferring special topological features, complementing the capabilities of salt templates.

### Freeze-drying method

Freeze-drying involves freezing the gel solvent and then reducing ambient pressure to remove solvent via sublimation [39]. Critical parameters—freezing direction, temperature, and duration—significantly influence solvent crystal growth orientation, rate, and spherulitic morphology, ultimately dictating the dried aerogel’s microstructure. Sublimation’s absence of capillary forces preserves the gel’s original network structure, a key reason for the technique’s widespread adoption.

The application of freeze-drying in SMPA fabrication can be logically categorized based on the polymer systems and technical innovations. This method is extensively used for fabricating SMPI aerogels. Typically, polyamide acid dissolved in water is freeze-dried to form a porous structure. Zhou et al. [40] synthesized distinct polyamides, copolymerized them, and freeze-dried the mixture to produce shape memory block polyimide (PI) aerogels; their mechanical and shape memory properties were tuned by varying component ratios. Building on this, Guo et al. [41] employed freeze-gelation, freeze-drying, and thermal annealing to fabricate SMPI/graphene aerogels (Fig. 3D), incorporating synthesized water-soluble polyacrylamide (PAA) into a graphene dispersion.

Beyond SMPIs, freeze-drying has been successfully extended to other SMPA systems, often with technical modifications. Li et al. [42] obtained chitosan (CS)/polyethylene glycol diacrylate (PEGDA) semi-interpenetrating network SMPAs via one-pot photopolymerization and freeze-drying. To achieve more ordered structures, Huang et al. [43,44] introduced unidirectional or bidirectional freezing techniques to induce oriented ice crystal growth, which—combined with low-temperature polymerization—enabled the creation of highly compressible and elastic PEGDA-based SMPAs reinforced with CNTs. In a different approach focusing on processability, Qi et al. [45] freeze-dried kneaded hydroxypropyl ethylcellulose/water/photoinitiator dough to form HPH (high molecular weight polyethylene glycol hydrogel) aerogels. Collectively, these studies underscore that freeze-drying serves as a versatile and indispensable strategy for constructing diverse SMPAs, with its parameters offering precise control over the microstructures that govern the final material properties.

### 4D printing method

4D printing is an additive manufacturing process that creates intelligent objects from stimuli-responsive materials. These objects can transform their shape or functionality over time when exposed to specific external stimuli. This technique can fabricate SMPs by integrating functional phases into the polymer matrix during printing, imparting multifunctional properties like light absorption and conductivity [46,47]. Distinguished by flexible, programmable manufacturing, it directly fabricates components from digital models without post-processing. To enable on-demand porous material fabrication, researchers employ direct writing technology, relying on precise viscosity control of functional phase inks. Consequently, current 4D printing advances for porous materials focus primarily on macrostructural design. Significant potential remains for achieving precise microstructural fabrication through printing parameter optimization. Despite challenges, this technology holds considerable promise for advancing porous material manufacturing.

In the 4D printing of PI-based aerogels, the research focus has been on balancing ink printability with final material performance. Geng et al. [48] fabricated highly recoverable PI/polyimide nanofiber (PIF) composite aerogels using 4D printing. They employed polyamide acid (PAA) as the matrix and PI nanofibers as rheological modifiers, preparing a precursor via direct writing. The composite aerogel was then synthesized through freeze-drying and stepwise thermal imidization (Fig. 3E). Fu et al. [49] developed shape memory PI/silica aerogel particle (PI/SAP) composite aerogels by formulating printing ink incorporating SAPs into PAA solution, followed by direct writing. The SAP effectively modulated the ink’s rheological behavior, ensuring printability. In the pursuit of novel macrostructures and properties, researchers have leveraged the superior design capabilities of 4D printing. Fan et al. [50] integrated the salt template method with 4D printing to fabricate shape memory SR (silicone rubber/polylactic acid)/PLA composite foams featuring concave hexagonal macropores and honeycomb-like microbubbles. The 4D-printed concave hexagonal macropores confer anisotropic negative Poisson’s ratio characteristics, overcoming the limitation of conventional foams unable to recover after large deformations. These works highlighted the great potential of 4D printing to transcend the limitations of traditional manufacturing and achieve customized mechanical properties.

### Other methods

Beyond conventional methods, emerging foaming techniques like supercritical CO₂, microwave, and laser foaming are gaining traction. Supercritical CO₂ foaming produces high-porosity foams with random pore distribution. This involves saturating a polymer matrix with supercritical CO₂—exhibiting gas-like diffusivity and liquid-like solubility—under controlled temperature/pressure. Inducing supersaturation via temperature increase or pressure decrease triggers CO₂ nucleation, growth, and eventual foam formation upon gas release [51]. You et al. [52] enhanced foaming performance in PVC (polyvinyl chloride)/polyurea systems, yielding SMPFs with superior expansion ratios and refined pores. Tian et al. [53] combined reactive plasticization, phase separation, and supercritical CO₂ foaming to fabricate SAN/PUA-T (styrene-acrylonitrile copolymer/polyurea) foams; microphase-separated pore walls endowed thermally triggered shape memory performance.

Zhang et al. [54] utilized microwave foaming for rapid processing, although resulting SMPFs displayed larger, nonuniform pores. Xiang et al. [55] pioneered laser foaming (Fig. 3F), generating microfoams in PLA via localized laser energy. This technique induces minimal PLA oxidation, preserving its intrinsic properties while enabling micropattern design applications. Du et al. [26] employed a composite foam method with divanillic acid-doped polycarbonate, creating foams that recover from compressed states to original shapes within 2 min.

A comparative summary of the primary fabrication strategies discussed above—gas foaming, template, freeze-drying, and 4D printing—is provided in Table 1. The table outlines key structural characteristics (e.g., typical pore size range and porosity connectivity) and process-related attributes (e.g., complexity and scalability) of each method. This comparison serves as a practical guide for selecting an appropriate fabrication strategy based on the target application’s requirements for pore architecture, structural order, and manufacturing considerations. The fabrication strategies of SMPPMs critically influence their microstructure formation. By selecting appropriate synthesis processes, researchers can manufacture SMPPMs with specific microstructures on demand. The gas foaming method is commonly employed to obtain open-cell structures, while the template method allows precise control over pore size. Through adjustment of freeze-drying parameters, SMPPMs with ordered and aligned architectures can be achieved. Additionally, 4D printing enables the fabrication of SMPPMs with customized microstructures based on digital models.

**Table 1.** Comparison of SMPPM fabrication strategies

Method	Microstructure features	Pore size	Porosity	Complexity	Scalability	Relative cost
Gas foaming	Disordered structure, mostly open-cell	Several μm to hundreds of μm	60–70%	Low (simple and convenient)	High	Low
Template method	Highly interconnected	Nanoscale to millimeter scale (dictated by template)	40–90%	Medium (involves template removal)	High (especially salt templating)	Low
Freeze-drying	Ordered structure, consecutive holes	Several μm to hundreds of μm (depends on ice crystals)	80–95%	High (requires controlled freezing)	Medium (limited by freeze-dryer size)	Medium
4D printing	Can be designed for specific connectivity	Primarily macro-architectural; micropore size depends on ink and parameters	40–80%	High (involves ink formulation and equipment operation)	Currently low, but high potential	High
Supercritical CO_2_ foaming	Mostly closed-cell	Micrometer to tens of micrometers	60–70%	High (requires precise pressure/temperature control)	High	Medium

## Designable Microstructures

Variations in preparation strategies generally influence the formation and development of the microporous structure in SMPPMs. The microstructural characteristics of SMPPMs play a decisive role in determining their shape memory behavior, mechanical performance, and functional adaptability. To achieve controlled design of porous topologies, researchers precisely regulate key process parameters such as phase separation conditions, types of pore-forming agents, and external field-induced factors. Based on the degree of structural order, the microstructures of SMPPMs can be broadly classified into 2 categories: ordered porous structures and disordered porous structures [56]. These distinct structural features directly influence the material’s shape recovery efficiency, cycling stability, and functional adaptability by modulating pore wall deformation mechanisms, transport pathways of external media, and energy dissipation modes. They provide a fundamental basis for structural design aimed at optimizing performance in specific application contexts.

### Disordered porous structures

Disordered porous SMPPMs exhibit isotropic behavior, classifiable into 3 architectures: open-cell, closed-cell, and mixed-cell structures. Open-cell networks feature interconnected channels enabling superior permeability, [57,58] critical for filtration and sound absorption. Closed-cell systems comprise sealed cavities leveraging gas pressure and elastic energy storage for enhanced mechanical cushioning. Mixed-cell designs synergize open/closed pores, balancing dynamic responsiveness and structural stability. These distinct architectures govern the shape memory performance through fundamentally different mechanisms. Open-cell structures accelerate stimulus (e.g., heat and solvent) diffusion throughout the network due to high interconnectivity, leading to faster and more uniform shape recovery. However, the thin pore walls and interconnected nature may compromise cyclic stability due to potential for plastic deformation or collapse under repeated loading [58]. Closed-cell structures amplify elastic recovery force through the entrapment of gas within sealed pores, which acts as a compressible spring. This results in higher recovery stress and improved energy dissipation [high SEA (specific energy absorption)], making them excellent for cushioning. The trade-off is slower stimulus response as the stimulus must permeate the solid cell walls, and recovery is often less complete if the gas pressure is not efficiently harnessed [59]. Mixed-cell structures enable a controlled balance between responsiveness and force. Open pores facilitate initial stimulus penetration, while closed pores contribute elastic energy and structural integrity. This allows for tunable strain energy release and a more balanced combination of recovery speed and recovery stress, mitigating the weaknesses of the pure architectures [30,60]. These architectures govern SMPPM recovery: Open pores accelerate stimulus diffusion, closed pores amplify elastic recovery, and mixed pores enable controlled strain energy release.

**Fig. 4.**
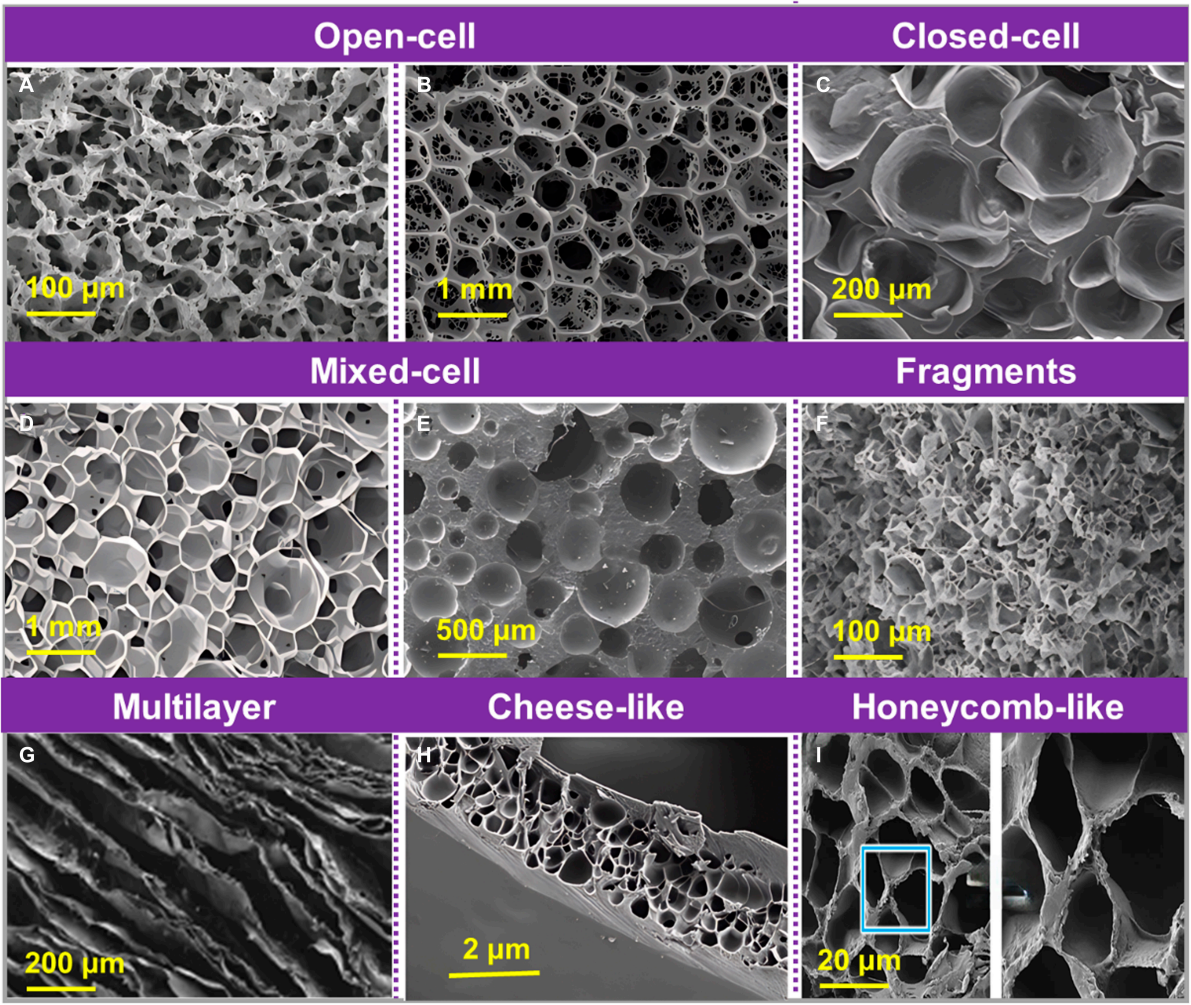
Microstructure of SMPPMs. (A and B) Open-cell structures. Reprinted with permission from [62, 63]. (C) Closed-cell structures. Reprinted with permission from [64]. (D and E) Mixed-cell structures. Reprinted with permission from [30, 66]. (F) Fragment structures. Reprinted with permission from [36]. (G) Multilayer structures. Reprinted with permission from [72]. (H) Cheese-like structures. 72. (I) Honeycomb-like structures. Reprinted with permission from [45].

Open-cell structures attract significant interest due to their high specific surface areas. Jia et al. [61] fabricated 3D interconnected aerogels via freeze-drying; incorporating 2 to 6 wt % Pt nanoparticles reduced pore size by 30%. The resulting finer and more interconnected pore network likely enhances the thermal conductivity and speed of thermal response, directly benefiting the shape recovery process. Shan et al. [62] constructed dual-scale TPU (thermoplastic polyurethane) foams by dispersing superhydrophobic SAPs, forming macropores and mesoporous walls (Fig. 4A). Zhou et al. [32] developed open-cell SMPFs via gas foaming, where nucleation, growth, and stabilization stages defined the architecture. Jungmann et al. [63] fabricated SMPF composite materials with open-cell structures via the impregnation method, which are characterized by high compressibility, as shown in Fig. 4B. Closed-cell structures excel in mechanical performance. Zhao et al. [59] synthesized epoxy-polyurethane foams with uniform spherical pores, thick walls, and 65.7% porosity, achieving ordered buckling deformation and a SEA of 7.65 J/g. Hu et al. [64] developed gradient phenolic foam; SiO₂/Al₂O₃ nanoparticles enhanced uniformity, compressive stress (35% to 50%), and thermal stability (Fig. 4C).

Previous studies have referred to porous structures with open and closed cell ratios ranging from 20% to 80% as mixed-cell structures [65]. Mixed-cell architectures enable advanced functional integration. Tian et al. [60] achieved expanded pores with 83.26% porosity and enhanced strength. Ren et al. [30] engineered gradient hybrid pores (0.38 to 8.27 mm) in PI/polyurethane foams, where macropores and closed cells synergistically improved low-frequency sound absorption via scattering and Helmholtz resonance (Fig. 4D). This multi-scale pore design demonstrates how mixed-cell structures can be tailored to optimize a specific functional output that is intrinsically linked to the shape memory matrix’s ability to undergo and recover from programmed strain. Zhou et al. [32] incorporated phosphorene nanosheets into polyaryloxyphosphonitrile foam, creating coexisting cells: Open cells hosted phase-change wax, while closed cells provided shape memory support for dual optical/thermal responsiveness. Luo et al. [66] developed hierarchical epoxy foams with micropores on macropore surfaces, significantly boosting heat transfer (Fig. 4E). The hierarchical design drastically increases the surface area for heat exchange, addressing a key limitation (slow heat transfer) in thick SMPPM samples and leading to more efficient and rapid shape recovery. Other disordered/hierarchical architectures include Hu et al.’s [36] fragment-like microporous materials enabling uniform stress distribution (Fig. 4F). Zhang et al. [67] integrated electro-spun PI nanofibers (micropores) with PI aerogel nanopores, forming interpenetrating micro/nano-networks. Liu et al. [68] deposited aerogel on MF scaffolds, creating dual-scaffold composites with superior compressive performance.

### Ordered porous structures

Ordered porous SMPPMs constitute ultralight materials defined by highly periodic pore architectures—cubic, hexagonal, or layered symmetries. Fabrication relies on template-directed assembly, self-assembly, or 4D printing, requiring precise control over precursor chemistry, template topology, phase separation kinetics, and interfacial forces during curing/drying. This enables exact regulation of pore size, morphology, connectivity, and long-range order. Key advantages include superior mass transfer efficiency, enhanced mechanical performance, and programmable functionality [69]. Consequently, these aerogels enable high-end applications like high-selectivity separation membranes, ultra-sensitive optical sensors, efficient catalytic reactors, and anisotropic thermal insulation, overcoming limitations of disordered counterparts.

The fabrication of ordered porous SMPPMs can be broadly classified based on the underlying pore-forming mechanism, with freezing-based techniques being the most prevalent. Within this category, distinct strategies have been developed to control ice crystal growth for tailoring the pore architecture. Huang et al. [43] employed bidirectional/unidirectional freezing–polymerization to create aerogels with long-range ordered architectures. Bidirectional freezing under horizontal/vertical temperature gradients produced 3D ordered channels featuring *XY*-plane honeycomb-like pores (50 to 100 μm). Unidirectional freezing yielded anisotropic aerogels with oriented 3D layered honeycomb pores—axial unidirectional channels and radial columnar layers—enabling superior radial stress distribution and resilience. Similarly, Zhang et al. [70] utilized directional freezing of a polyamic acid/CNT solution, where the CNTs acted as crosslinkers and the freezing process created 3D interconnected pores. They demonstrated that increasing CNT content could suppress ice crystal growth and reduce pore size. Liu et al. [71] applied freeze-spinning with a rotating spool in liquid nitrogen to construct anisotropic ordered porous PVA materials. Radial/multi-directional ice crystal growth forced PVA molecules into interstitial spaces, forming dendritic radial arrangements of ordered channels. Zhou et al. [40] fabricated SMPI aerogels via freeze-drying, achieving uniform ordered 3D porous structures that synergistically preserved high tensile modulus while improving shape fixation/recovery. Collectively, these freezing-based methods highlight the critical role of controlling ice crystal morphology and growth direction as a versatile pathway to achieving highly ordered and often anisotropic porous structures.

Alternative template-based and processing-driven methods offer complementary routes to ordered porosity. Li et al. [72] combined bubble-templating with freeze-drying to fabricate shape memory hierarchical PI aerogels exhibiting multilayered and cheese-like microstructures (Fig. 4G and H). Dynamic reversible physical entanglements between molecular chains impart molecular-level flexibility. Qi et al. [45] created hydroxypropyl ethylene glycol (HPEG)-based ordered porous aerogels (HPAs) using kneading–folding-assisted ultraviolet (UV) crosslinking. HPA exhibits a highly ordered 3D honeycomb-like macroporous architecture with smooth, periodically arranged walls (Fig. 4I). These studies successfully generated ordered pores and provide distinct microstructural features.

## Multi-Temperature Responsive SMPPMs

The fundamental properties of SMPPMs, such as their chemical stability, thermal stability, and environmental resistance, are predominantly governed by the molecular structure and types of chemical bonds present in their matrix material. At the same time, the shape transition temperature—serving as a key parameter of the shape memory effect—is closely associated with the glass transition temperature (*T*_g_) of the matrix material. *T*_g_ determines the threshold for significant changes in the mobility of polymer chain segments, controls the temperature conditions for the fixation and restoration of the temporary shape during the shape memory process, and thereby determines the shape transformation temperature of SMPPMs. This relationship directly influences the activation conditions and defines the applicable scenarios for the shape memory functionality of SMPPMs. Precise selection and design of the matrix enable targeted design and regulation of the performance and functionality of SMPPMs. Based on the shape transformation temperature of porous materials, researchers have developed low-temperature responsive SMPPMs (shape transformation temperature is below 60 °C), medium-temperature responsive SMPPMs (shape transformation temperature range is from 60 to 120 °C), and high-temperature responsive SMPPMs (shape transformation temperature above 120 °C). SMPPMs with different response temperatures are fabricated from different polymer matrices using pore-forming techniques, as shown in Table 2. This *T*_g_-based categorization not only helps in selecting the appropriate material for a specific application temperature but also frames the following detailed discussion on the distinct polymer matrices, unique properties, and primary application fields characteristic of each temperature class.

**Table 2.** SMP matrices, strategies, and response temperature of SMPPMs

SMP matrices	Strategies	Response temperature	Reference
Shape memory polyurethane	Impregnation method	37 °C	[73]
	Impregnation method	40 °C	[75]
	Impregnation method	37 °C	[76]
	Impregnation method	70 °C	[77]
	Supercritical CO_2_ foaming	40 °C	[78]
	Supercritical CO_2_ foaming	42 °C	[79]
Shape memory polycaprolactone	Microwave foaming	43.6–55.2 °C	[54]
	Gas foaming	54 °C	[82]
Shape memory epoxy	Gas foaming	91 °C	[60]
	Gas foaming	80 °C	[63]
Shape memory polyvinyl alcohol	Template method	80 °C	[87]
Shape memory polyimide	Freeze-drying	230 °C	[92]
	Freeze-drying	310 °C	[68]
	Freeze-drying	280 °C	[41]
	4D printing	240 °C	[49]
Shape memory phenolic resin	Gas foaming	127 °C	[118]
	Template method	90–170 °C	[35]
Shape memory phthalimide resin	Template method	350 °C	[36]

### Low-temperature responsive SMPPMs

Low-temperature responsive SMPPMs, with shape memory transition typically below 60 °C, are ideal for biomedical applications due to their low activation temperature (near body temperature), excellent biocompatibility, and potential biodegradability. Precise tuning of *T*_g_, recovery rate, and mechanical strength are achievable through molecular structure modification.

**Fig. 5.**
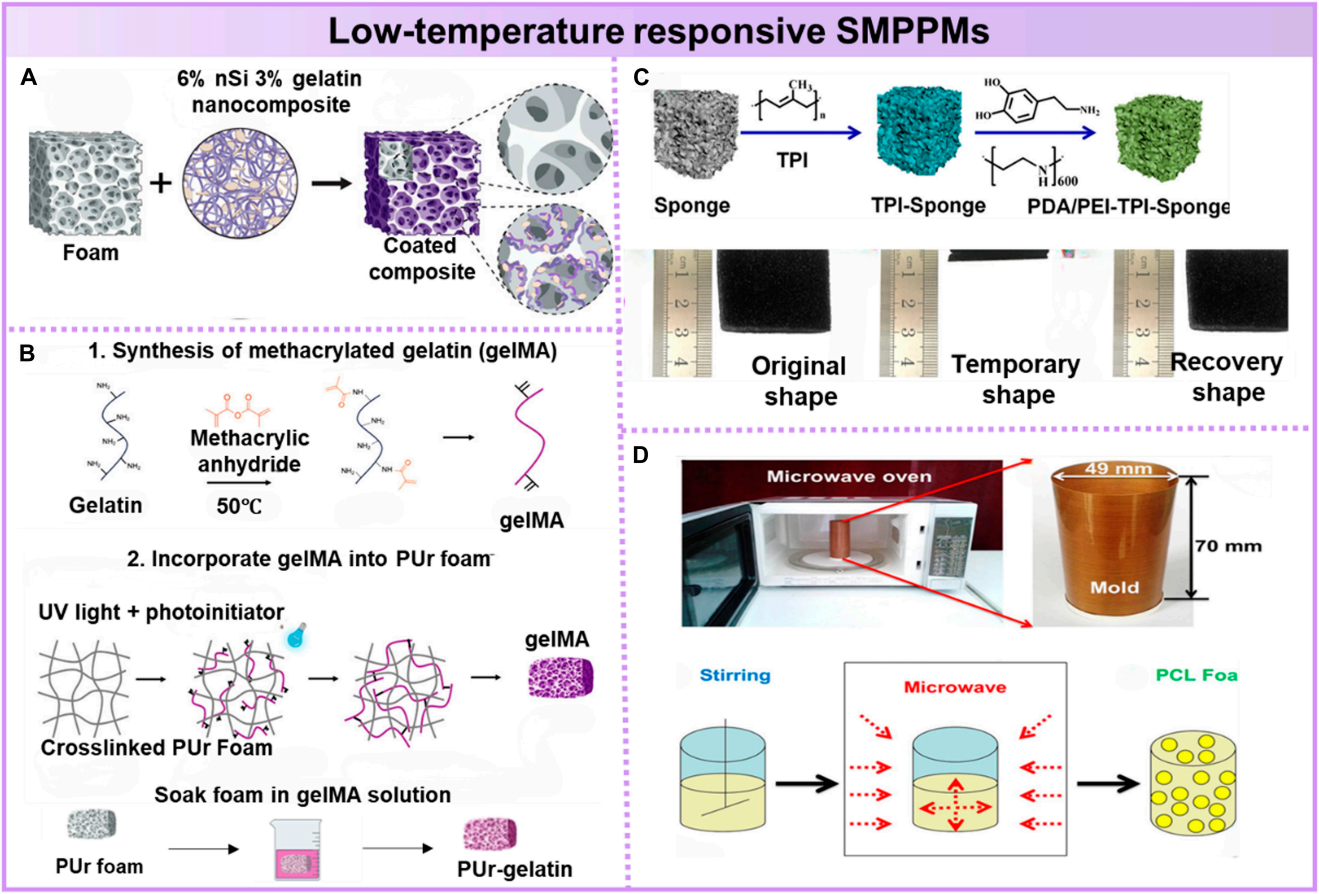
Low-temperature responsive SMPPMs. (A) Design and preparation of SMPU foams. Reprinted with permission from [73]. (B) Design and preparation of SMPU composites. Reprinted with permission from [76]. (C) Design of PDA/PEI-TPI-Sponge (plyophilic polydopamine/polyethylenimine-trans-1,4-polyisoprene-Sponge). Reprinted with permission from [77]. (D) Preparation of shape memory polycaprolactone foam by microwave foaming. Reprinted with permission from [54].

SMPU-based porous materials attract attention for their biocompatibility, high recovery force, and processability. While early isocyanate–polyol–water systems yielded unstable foams, Hargett et al. [73] innovated using hexamethylene diisocyanate, triethanolamine, and hydroxypropyl ethylenediamine with 0.6 wt % foaming agent, producing macroporous foam (Fig. 5A). Kim et al. [74] fabricated dual-pore-size foams via salt templating, demonstrating that reduced pore size lowers thermal conductivity while enhancing mechanical performance.

In the context of impregnation-based approaches, Zhu et al. [75] developed ultra-lightweight foam with a d-glucuronic acid-CS/SWCNT (single-walled carbon nanotube) layer and paraffin layer, achieving 56-dB electromagnetic shielding and strain adjustability. Petryk et al. [76] incorporated collagen/gelatin into PU (polyurethane) foam (Fig. 5B), significantly improving shape retention. Liu et al. [77] deposited trans-polyisoprene and polydopamine/polyethyleneimine onto the foam surface (Fig. 5C), imparting superhydrophilicity and temperature-regulated reversible liquid absorption at 70 °C. These studies collectively highlight a trend in SMPU foam research beyond mere shape memory, moving toward sophisticated structural design and surface engineering for added functionalities like sensing and liquid control.

In the realm of SMPU aerogels, a clear developmental trajectory exists. SMPU aerogel research originated with Donthula et al. [78], who synthesized the first SMPA via triisocyanate-diol sol–gel reaction and supercritical drying, achieving tunable properties and up to 98% recovery. Malakooti et al. [79] utilized similar raw materials to fabricate aerogels with a negative Poisson’s ratio, enabling programmable deformation at temperatures above 30 °C. Doulah et al. [80] accelerated gelation using metal salt catalysts, observing increased porosity as microstructure evolved from bi-continuous to spherulitic/microspherical forms.

In the field of low-temperature responsive SMPPMs, significant advancements have been achieved beyond polyurethane-based systems [42,81]. Zhang et al. [54] employed microwave-assisted rapid foaming technology to fabricate biodegradable crosslinked polycaprolactone foam, demonstrating that increased crosslinking agent content lowers the material’s *T*_g_ and quantifying the differences in shape recovery rates among various actuation mechanisms, as illustrated in Fig. 5D. Sauter et al. [82] utilized a poly(ω-pentadecalactone)-poly(ε-caprolactone) block copolymer as the base material and produced anisotropic foams through supercritical CO₂ processing. Micro-computed tomography analysis revealed a single-cell shape anisotropy ratio of 1.72, with a longitudinal-to-transverse Young’s modulus ratio of 4.3, indicating the potential for structure-directed regulation of mechanical properties. Ma et al. [83] integrated physical foaming with freeze-drying to develop PVA/phase-change microcapsule composite porous materials. Their results showed that increasing the microcapsule content enhances phase-change energy storage capacity, but concurrently reduces pore density, average pore size, and water absorption rate.

### Medium-temperature responsive SMPPMs

The shape memory transformation temperature of medium-temperature responsive SMPPMs typically falls within the range of 60 to 120 °C, accompanied by favorable thermal stability. Predominantly based on epoxy resin systems, these materials are particularly suited for deployable structural applications in the aerospace industry. Their key advantages include high shape recovery accuracy, excellent environmental stability, and tunable actuation temperatures, which collectively enable reliable deformation performance under extreme thermal fluctuations commonly encountered in space environments [84]. Moreover, certain other medium-temperature responsive SMPPM systems demonstrate applicability in auxiliary fields such as noise reduction and water treatment.

**Fig. 6.**
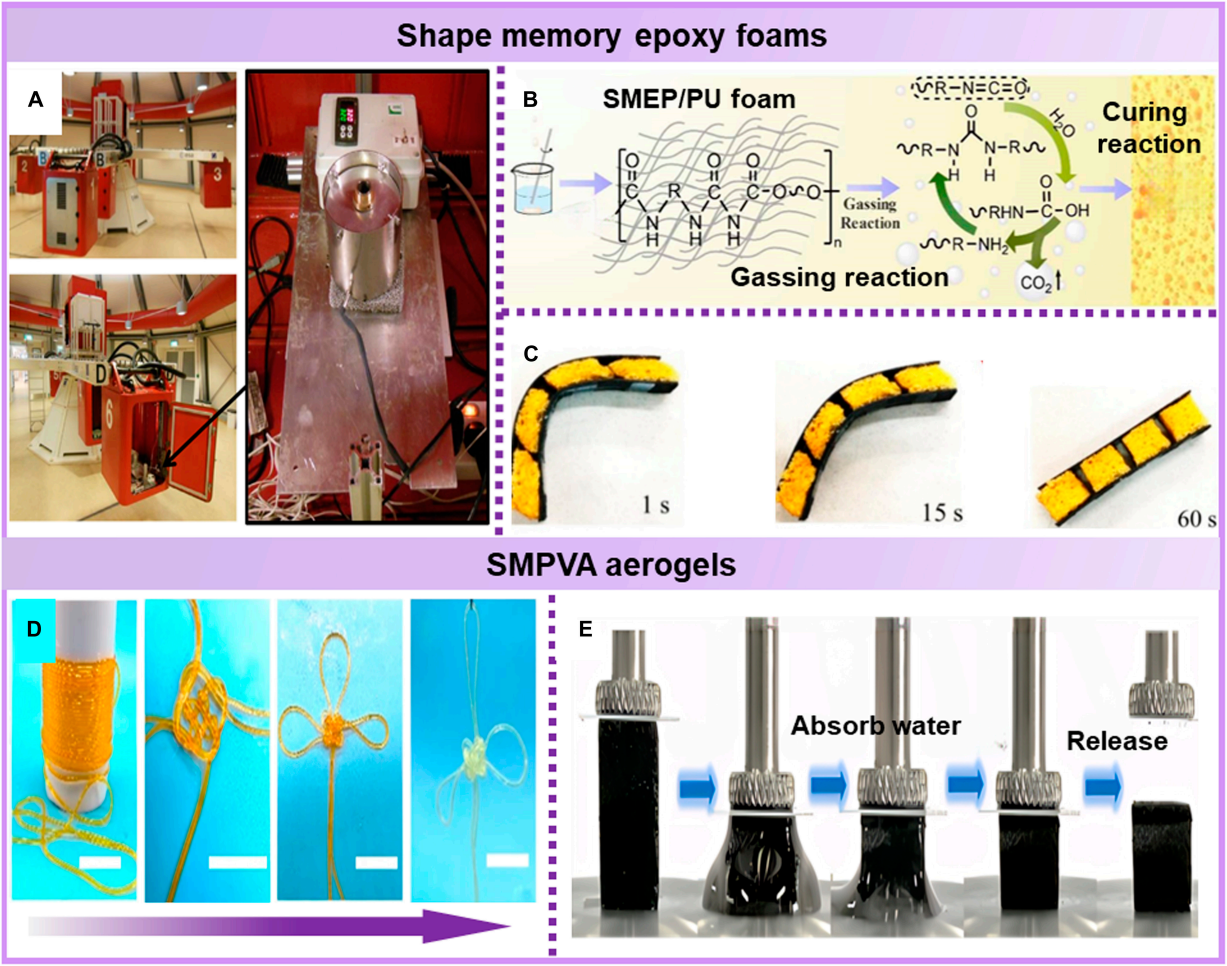
Medium-temperature responsive SMPPMs. (A) Preparation of SMEP foams in microgravity and hyper-gravity environments. Reprinted with permission from [85]. (B) Design of shape memory EP (epoxy)/PU foam composite materials. Reprinted with permission from [66]. (C) Shape recovery process of SMEP foam sandwich panels. Reprinted with permission from [59]. (D) Programmable shape memory PVA aerogel. Reprinted with permission from [86]. (E) Shape memory PVA aerogel with shape recovery by water absorption. Reprinted with permission from [89].

Epoxy-based SMPPMs serve as a representative class of medium-temperature responsive systems and allow precise control of the *T*_g_ through the selection and proportion of curing agents. Microgravity experiments conducted on the BION-M1 spacecraft launched by the Soyuz-2 rocket confirmed that the shape recovery ability of epoxy foam is not affected by microgravity conditions, expanding the scope of SMEP foam applications in space. Some researchers prepared foam using solid-state foaming in simulated microgravity and hyper-gravity environments (Fig. 6A), revealing for the first time the dual influence of the gravitational vector on the foaming process: The movement of the flow front promotes foam expansion, whereas poor thermal contact between the sample and oven exerts an inhibitory effect [85]. Collectively, these studies establish a comprehensive technological framework spanning constitutive modeling, functional design, process innovation, and space application validation, positioning epoxy-based SMPPMs as key candidate materials for intelligent aerospace structural systems.

Concurrently, significant efforts have been directed toward developing functional epoxy-based structures with enhanced performance. Leng’s research group has achieved a series of advancements in polyurethane/epoxy composite systems: Luo et al. [66] applied a similar methodology to produce epoxy foam (Fig. 6B), which was validated for geostationary orbit adaptability through space environment simulation, including thermal equilibrium testing and thermal vacuum cycling. Subsequently, Zhao et al. [59] further utilized this epoxy foam as the core material and carbon fiber-reinforced shape memory polymer composite (SMPC) as the face sheets to construct a shape memory sandwich structure (Fig. 6C). Compressing the core material by 60% resulted in a 232% increase in peak impact contact force and a 40% improvement in kinetic energy decay rate, offering an innovative solution for deployable aerospace structures. Beyond epoxy resins, medium-temperature SMPPMs exhibit diversified development, particularly with PVA-based aerogels, which are valued for their superhydrophilicity and ease of functionalization. PVA-based aerogels have attracted significant attention for functionalization due to their inherent superhydrophilicity, derived from hydroxyl group content. Li et al. [86] innovatively synthesized organic gels via reactions between ANF (aramid nanofibers) and PVA, and integrated solvent exchange with freeze-drying and physical programming to fabricate shape memory aerogels capable of repeatable stretching, bending, and twisting, as illustrated in Fig. 6D. Wang et al. [87] prepared a PVA matrix using a pre-crosslinked ice template method and subsequently surface-loaded it with COF-1/BiPO₄ photocatalysts, enabling recyclable degradation of tetracycline pollutants. Mohsenian et al. [88] enhanced the crosslinking density of the PVA network and imparted shape memory functionality through Fe^3+^/Cu^2+^ ion crosslinking. Yu et al. [89] combined carboxylated carbon nanotubes and graphene with PVA to fabricate a multi-layer gradient-structured aerogel via freeze-drying (Fig. 6E), which exhibited exceptional durability under 2,000 cycles of 90% strain in aqueous environments and demonstrated noise-reduction capabilities.

### High-temperature responsive SMPPMs

To expand the application of SMPPMs in high-temperature environments, researchers have selected resin matrices with superior thermal performance to develop SMPPMs. High-temperature responsive SMPPMs are primarily represented by systems such as phenolic resins, PI, polyureas, and phthalocyanine nitriles, with shape transformation temperatures typically exceeding 120 °C. These materials are particularly suitable for extreme environmental designs in the aerospace industry. They combine excellent high-temperature stability—maintaining structural integrity at temperatures above 350 °C for extended periods—with strong ablation resistance, enabling them to withstand thermal shocks and high-speed particle erosion during atmospheric reentry. These properties make them promising candidates for critical thermal structural components, such as rocket engine thermal protection layers and spacecraft heat shield tiles, offering lightweight and intelligent thermal management solutions for hypersonic vehicles.

PI-based SMPPMs represent a typical class of high-temperature SMPPMs [90,91], with research focusing on molecular design, structural regulation, and functional composite synergies. Li et al. [92] integrated molecular structure optimization with ice template technology and found that freezing at −196 °C significantly enhances ice crystal nucleation while suppressing crystal growth, resulting in a dense framework with fine pore structures. This contributes to improved thermodynamic and compressive properties of the aerogels. The physical cross-linking network formed by interchain interactions in PI provides the structural basis for the stability of the porous architecture. To address the key challenges of limited resilience and slow recovery rates, Zhou et al. [40] and Li et al. [72] emphasized, from the perspective of cross-linking network design, that performance improvements can be achieved through composite reinforcement strategies.

**Fig. 7.**
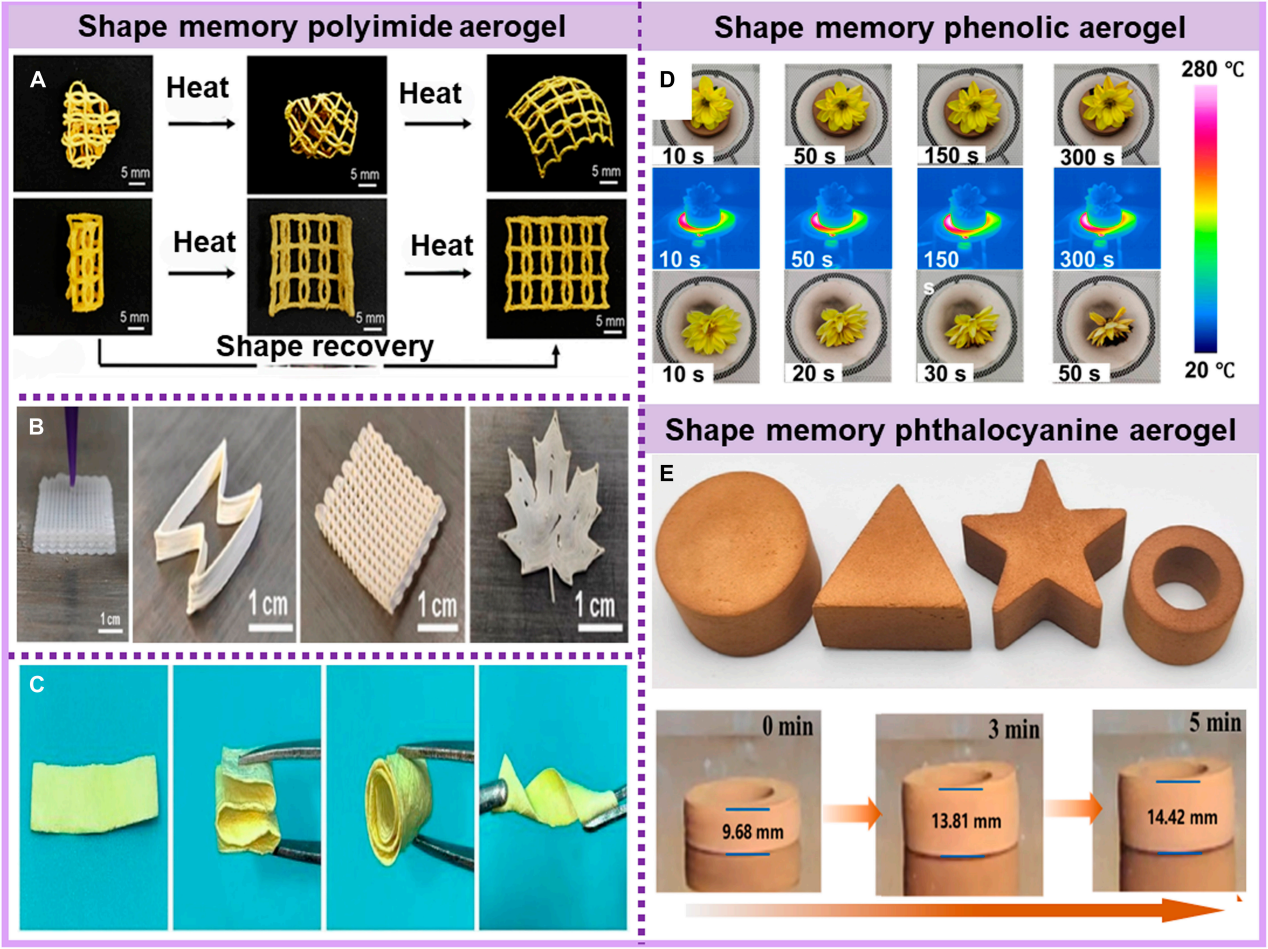
High-temperature responsive SMPPMs. (A) Shape recovery process of SMPI aerogel. Reprinted with permission from [48]. (B) SMPI aerogel fabricated by 4D printing. Reprinted with permission from [49]. (C) Foldable, twistable flexible SMPI aerogel. Reprinted with permission from [67]. (D) Shape memory phenolic aerogel with tunable thermal insulation properties. Reprinted with permission from [35]. (E) Easily machinable phthalic nitrile aerogel. Reprinted with permission from [36].

Functional fillers have become a critical approach for enhancing shape recovery performance in SMPPMs. Guo et al. [41] fabricated a PI/graphene aerogel, achieving significantly elevated recovery stress. Geng et al. [48] employed inkjet printing technology to produce composite materials with a hyperbolic surface structure (Fig. 7A), which markedly enhanced programmable recovery stress. Fu et al. [49] synthesized a PI/SAP, in which the incorporation of silica improved mechanical strength and provided ablation resistance, as illustrated in Fig. 7B. Zhang et al. [70] introduced functionalized carbon nanotubes into the polymer matrix and fabricated aerogels using a directional freeze-drying method. The carbon nanotubes enhanced intermolecular interactions, thereby improving both recovery stress and thermal conductivity, with a reported shape recovery rate of 97.2%. These studies collectively establish filler reinforcement as a versatile method for enhancing the functional performance of PI-based SMPPMs.

To simultaneously address the challenges of insufficient mechanical strength and slow recovery rate, Zhang et al. [67] proposed an innovative PI fiber reinforcement strategy. In this approach, pre-prepared nanofibers were immersed in a PI solution, followed by a gel–sol reaction, solvent exchange, and supercritical CO₂ drying, resulting in an aerogel with a uniformly reinforced fibrous framework (Fig. 7C). Collectively, these studies establish a comprehensive technological framework spanning microstructural regulation, functional composite design, and fiber reinforcement, thereby advancing the development of PI-based SMPPMs.

In the context of PI systems, research on other high-temperature responsive SMPPMs has also been reported. Leng’s research group has developed high-temperature SMPPMs specifically tailored for aerospace applications. Hu et al. [64] fabricated shape memory phenolic foam with an open-cell structure using *n*-pentane as a blowing agent. This foam effectively addresses the inherent brittleness and foaming process control challenges associated with traditional phenolic foam. The presence of benzene rings in the phenolic resin matrix imparts excellent thermal stability and enables multidimensional shape memory behavior, opening new possibilities for the application of phenolic resin materials in advanced functional systems. In subsequent studies, Hu et al. [35] further developed shape memory phenolic materials with enhanced high-temperature resistance and ablation resistance, achieving a linear ablation rate below 0.01 mm/s. Additionally, shape memory phenolic aerogels were synthesized using a template-based approach, demonstrating superior shape memory performance. These aerogels allow for controlled thermal insulation properties through microstructural and thickness modulation based on shape memory characteristics, as illustrated in Fig. 7D. Moreover, Hu et al. [36] reported the development of SMPNAs with both ablation resistance and high-temperature actuation capabilities. These aerogels can be processed into various shapes and exhibit favorable processability, as illustrated in Fig. 7E. Upon treatment at 800 °C, SMPNAs undergo carbonization and display electrical conductivity, demonstrating excellent broadband electromagnetic absorption properties. These characteristics suggest promising applications in thermal insulation and infrared stealth technologies.

Research on other polymer systems further demonstrates the diversity of high-temperature SMPPMs. Xia et al. [93] synthesized Eucommia gum foam through a combined process of chemical crosslinking and chemical foaming. This integrated methodology significantly improved pore uniformity, achieving a shape retention rate of 94.43% and a full shape recovery rate of 100%. In the field of polyurea-based systems, efforts have focused on high-performance composites. Wang et al. [94] and Zhao et al. [51] employed plasticization strategies to process high-performance polymers like PPO [poly(phenylene oxide)] and PAEK [poly(aryl ether ketone)] into shape memory foams, retaining inherent heat and flame resistance. These efforts on alternative polymers highlight the ongoing exploration of new material platforms to meet the specific demands of high-temperature applications.

## Stimuli-Responsive Properties

Similar to SMPs, SMPPMs also exhibit a variety of actuation mechanisms, including thermal, electrical, optical, magnetic, microwave, and solution-driven actuation. For SMPs, the prerequisite for shape memory recovery is reaching the *T*_g_, which makes thermal actuation the most fundamental and widely utilized stimulation mechanism for SMPPMs. In recent years, researchers have successfully imparted photo-responsive, electro-responsive, magneto-responsive, and even multi-stimuli responsive capabilities to SMPPMs by incorporating functional fillers into the polymer matrix. The choice of actuation method is critical for application performance, as each mechanism offers distinct advantages and limitations in terms of response speed, spatial control, energy efficiency, and operational complexity.

Thermal actuation is universal and easy to implement via hot air or liquids, suited for bulk recovery where localization is not needed. However, it suffers from slow response due to low thermal conductivity and poor spatial control. Light actuation enables remote, precise, and rapid stimulation, ideal for noncontact applications like biomedicine and soft robotics. Its drawbacks include limited penetration depth, potential thermal damage, and the need for photothermal fillers that may affect properties. Electrical actuation offers on-demand, efficient Joule heating with fast response and easy integration into electronics, making it suitable for wearables and sensors. Yet, it requires conductive fillers—adding cost and complexity—and raises safety concerns such as overheating or short circuits. Magnetic actuation allows deep penetration and high-speed heating but requires uniform dispersion of magnetic particles. Microwave heating enables volumetric and rapid thermal energy transfer, yet requires specialized equipment and compatible dielectric properties. Solvent-induced actuation is simple and efficient for specific chemical environments but lacks generality and may provoke swelling or structural degradation. The selection of an actuation strategy should therefore be guided by the application context, considering constraints related to stimulus accessibility, material compatibility, spatial resolution, and safety requirements. The following sections detail recent advances within each stimulation modality.

### Thermal-responsive

The majority of SMPPMs can undergo shape memory cycles upon thermal stimulation. The advantages of thermally driven SMPPMs lie in the diversity of available heating methods, which can be implemented using various sources such as hot water, hot air, and other thermal media. Although the porous structure of SMPPMs may reduce their thermal conductivity, the shape memory performance, which is governed by the molecular crosslinking network of the material, remains unaffected. Elevating the ambient temperature of SMPPMs can still effectively facilitate the shape recovery process.

**Fig. 8.**
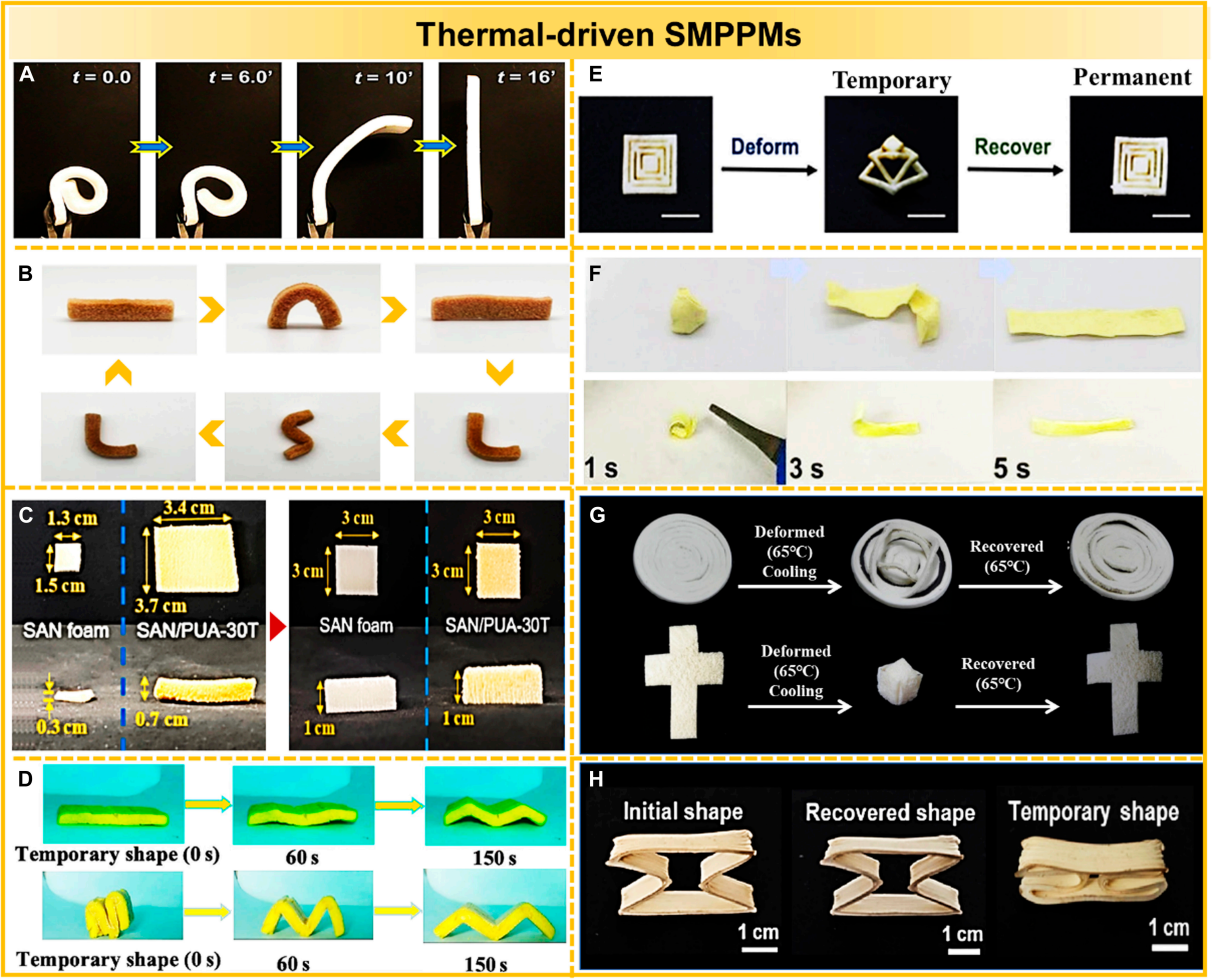
Thermal-driven SMPPMs. (A) Thermal-driven shape recovery process of the first SMPA. Reprinted with permission from [78]. (B) Thermal-driven shape recovery process of SMPF based on tung oil. Reprinted with permission from [60]. (C) Thermal-driven shape recovery process of the SMPU foam. Reprinted with permission from [53]. (D) Thermal-driven shape recovery process of shape memory phenolic foam. Reprinted with permission from [64]. (E to G) Thermal-driven shape recovery process of programmable SMPPMs. Reprinted with permission from [42, 67, 95]. (H) Thermal-driven shape recovery process of 4D-printed SMPPMs. Reprinted with permission from [49].

As illustrated in Fig. 8A, the SMPI aerogels reported by Donthula et al. [78] can recover from a bent temporary shape to their original straight configuration within 16 s. The tung oil-based SMPFs prepared by Tian et al. [60] exhibit triple shape memory behavior, as shown in Fig. 8B. The SMPU foam developed by Tian et al. [53] can be compressed from 0.8 cm to 0.3 cm and fully recover in a 200 °C environment. Due to the porous structure, the recovery speed is relatively slow, requiring approximately 10 min to complete the entire process (Fig. 8C). The material recovers from a U-shape to a rectangular shape at 120 °C, and after dynamic rearrangement of ester bonds at 160 °C, it can also recover from an S-shape to an L-shape. The shape memory phenolic foam developed by Hu et al. [64] demonstrates multidimensional shape recovery capabilities (Fig. 8D), showcasing excellent performance under thermal stimulation.

The SMPFs fabricated by Chen et al. [95] using a 2-step curing strategy can be programmed from a 2D shape into a 3D pyramid configuration and recover under thermal activation (Fig. 8E). The SMPI aerogel composite developed by Zhang et al. [67] exhibits remarkable flexibility, enabling it to be twisted from a flat sheet into irregular spherical and other 3D forms, with full shape recovery achieved within 5 s (Fig. 8F). The SMPPMs developed by Zhou et al. [32] and Zhou et al. [40] also demonstrate 3D shape recovery capabilities. The SMPAs prepared by Li et al. [42], as shown in Fig. 8G, can similarly undergo 3D shape recovery. Moreover, this aerogel is capable of surface morphology deformation, revealing preset Chinese character information upon heating to the shape transition temperature. Fu et al. [49] have further advanced the field by fabricating SMPI aerogels into 3D foldable structures via 4D printing, which can complete shape recovery at 250 °C (Fig. 8H).

Thermal actuation presents significant challenges for SMPPMs. The porous and discontinuous structure of SMPPMs results in inherently low thermal conductivity, leading to prolonged response times during thermal actuation compared to other continuous SMP configurations—particularly affecting SMPAs. To address this limitation, researchers have primarily adopted 2 strategies. The first strategy involves incorporating thermally conductive fillers with high thermal conductivity, such as graphene, CNTs, or carbon black, into the SMP matrix to fabricate porous shape memory materials. While this approach enhances thermal conductivity, it requires precise control over the uniform dispersion of the filler within the polymer matrix to ensure optimal performance.

Zhang et al. [70] demonstrated that the incorporation of CNTs into SMPAs significantly increased the aerogel’s actuation force, thereby improving its shape recovery rate. The second strategy focuses on constructing internal thermal conduction networks within the SMP structure to reduce actuation response times. Recent advancements have involved immersing thermally conductive porous materials, such as carbon foam, into SMP solutions to fabricate SMPFs. This method not only enhances the thermal conductivity of SMPFs but also imparts dual electro-thermal actuation capabilities. Wang et al. [96] developed a 3D network rGO/SMPU/PU foam through solution polymerization and chemical self-assembly. The synergistic interaction among the highly elastic PU foam framework, thin SMPU layer, and highly thermally conductive reduced graphene oxide (rGO) layer significantly improved the thermal conductivity of SMPFs, reducing their response time to less than 1 s. Furthermore, these SMPFs exhibit excellent mechanical properties and long-term cyclic stability, demonstrating considerable potential for applications in intelligent information storage and responsive materials.

### Light-responsive

**Fig. 9.**
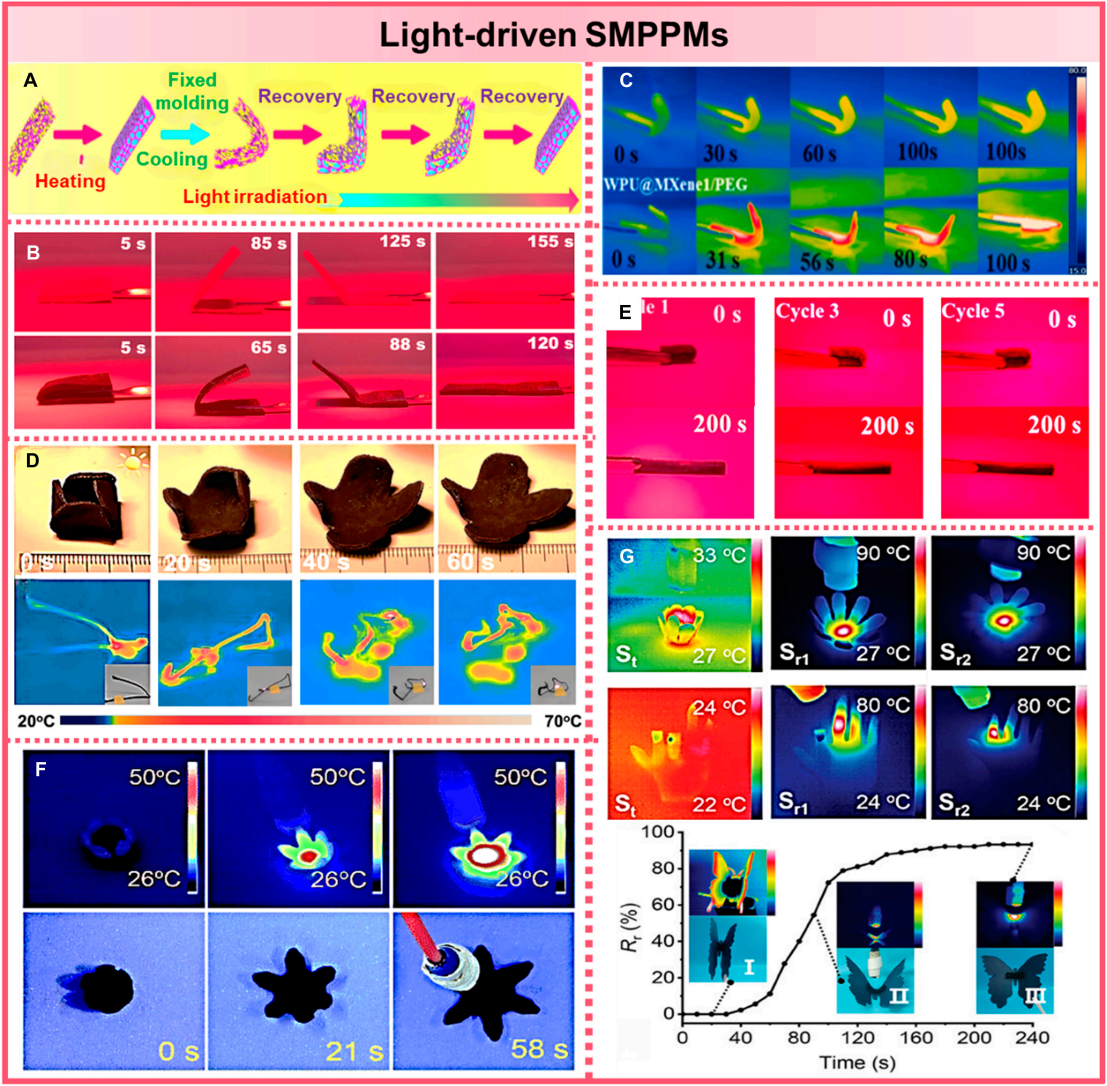
Light-driven SMPPMs. (A) Schematic of the light-driven response process of SMPPMs. Reprinted with permission from [99]. (B) Light-driven shape recovery process of shape memory PDAP (polyaryloxyphosphazene) foams and their composites. Reprinted with permission from [32]. (C) Light-driven shape recovery process of the shape memory PPEG (polyethylene glycol)/MXene foam composites. Reprinted with permission from [100]. (D) Light-driven shape recovery process of shape memory PPHPA (high molecular weight polyethylene glycol hydrogel)/Ni-HITP aerogel composites. Reprinted with permission from [45]. (E) Light-driven shape memory recovery process of SMPF in 5 cycles. Reprinted with permission from [101]. (F) Light-driven shape recovery process of shape memory PEGDA/CNT aerogel composites. Reprinted with permission from [43]. (G) Light-driven shape recovery process of shape memory PEGDA/ MXene aerogel composites. Reprinted with permission from [44].

Light-driven actuation provides precise and rapid partial shape recovery for SMPPMs, contrasting with conventional thermal-driven methods that require uniform heating. By adjusting irradiation position, duration, and intensity, light enables localized recovery with controlled degrees and rates [97,98]. The porous structure of SMPs impairs thermal conductivity, preventing heat transfer to non-illuminated areas and confining shape transition temperatures exclusively to irradiated zones for selective recovery. Current light-driven SMPs fall into 2 categories: those with intrinsic photothermal capabilities achieved by integrating functional groups into the polymer matrix via synthesis, where UV irradiation converts light to heat to trigger recovery, and extrinsic composites incorporating high-efficiency photothermal phases into nonresponsive SMP matrices to confer photo-actuation properties. Figure 9A illustrates the shape memory mechanism of the modified light-driven SMPPMs [99]. Under light irradiation, these materials absorb optical energy, convert it into thermal energy, and thereby increase in temperature. When the temperature reaches the shape transition threshold, the material gradually recovers to its original shape.

A significant portion of research focuses on carbon-based nanomaterials as highly efficient photothermal fillers, with studies exploring graphene, CNTs, and MXene. As shown in Fig. 9B, Zhou et al. [32] incorporated graphene into polyaryletherphosphazene foam to fabricate SMPF composites with photothermal conversion capabilities. These composites can complete the shape recovery process within 40 s under near-infrared lamp irradiation. Compared to the material without graphene, the response time is significantly shortened. Hu et al. [100] introduced MXene into waterborne polyurethane and subsequently compounded it with polyethylene glycol to develop shape memory composites exhibiting both photothermal conversion and energy storage properties (Fig. 9C). The authors observed that the shape recovery speed is positively correlated with the MXene content. When the doping level reaches 5 wt %, the composite achieves full shape recovery within 78 s.

Qi et al. [45] modified phase-change materials with organometallic frameworks to fabricate shape memory HPA/Ni-HITP (hexaiminotriphenylene) aerogel composites (Fig. 9D). Apart from their light-driven shape recovery properties, these materials can achieve selective shape recovery at specific locations through light irradiation. The SMPFs developed by Wu et al. [101] demonstrate excellent shape memory performance under light irradiation (Fig. 9E). In 5 consecutive shape memory cycles, complete shape recovery is achieved each time. In another study by Huang et al. [43], ammonia-treated CNTs were incorporated into PEGDA to prepare SMPA composites. Upon reaching a surface temperature above 50 °C under near-infrared irradiation, the material completes the 3D shape recovery process within 58 s (Fig. 9F). In a follow-up study, Huang et al. [44] employed a similar methodology to incorporate MXene into PEGDA, resulting in SMPA composites with multi-stimuli responsive behavior. Under near-infrared light of varying intensities, the material can transition from temporary shapes (e.g., butterfly and flower bud configurations) to its original shape (Fig. 9G).

### Electricity-responsive

Most reported SMPs lack inherent conductivity, limiting their applicability. To expand actuation methods, conductive fillers (e.g., graphene, carbon nanofibers, and metals) are incorporated into polymer matrices, endowing electrical conductivity [102]. Electrically driven SMP composites utilize Joule heat from applied currents. The fillers’ continuous conductive network mitigates SMPPMs’ inherent poor thermal conductivity. Advantages include eliminating external heat sources and enabling efficient internal heating via electrothermal conversion, offering high efficiency with minimal energy loss. However, practical considerations require attention: Driving voltage must be controlled to prevent rapid overheating that could trigger thermal decomposition and combustion; rational conductive pathways must ensure smooth, continuous circuits to enable fast recovery while avoiding short circuits and safety risks; the polymer matrix must possess high thermal stability and flame retardancy to ensure operational safety.

**Fig. 10.**
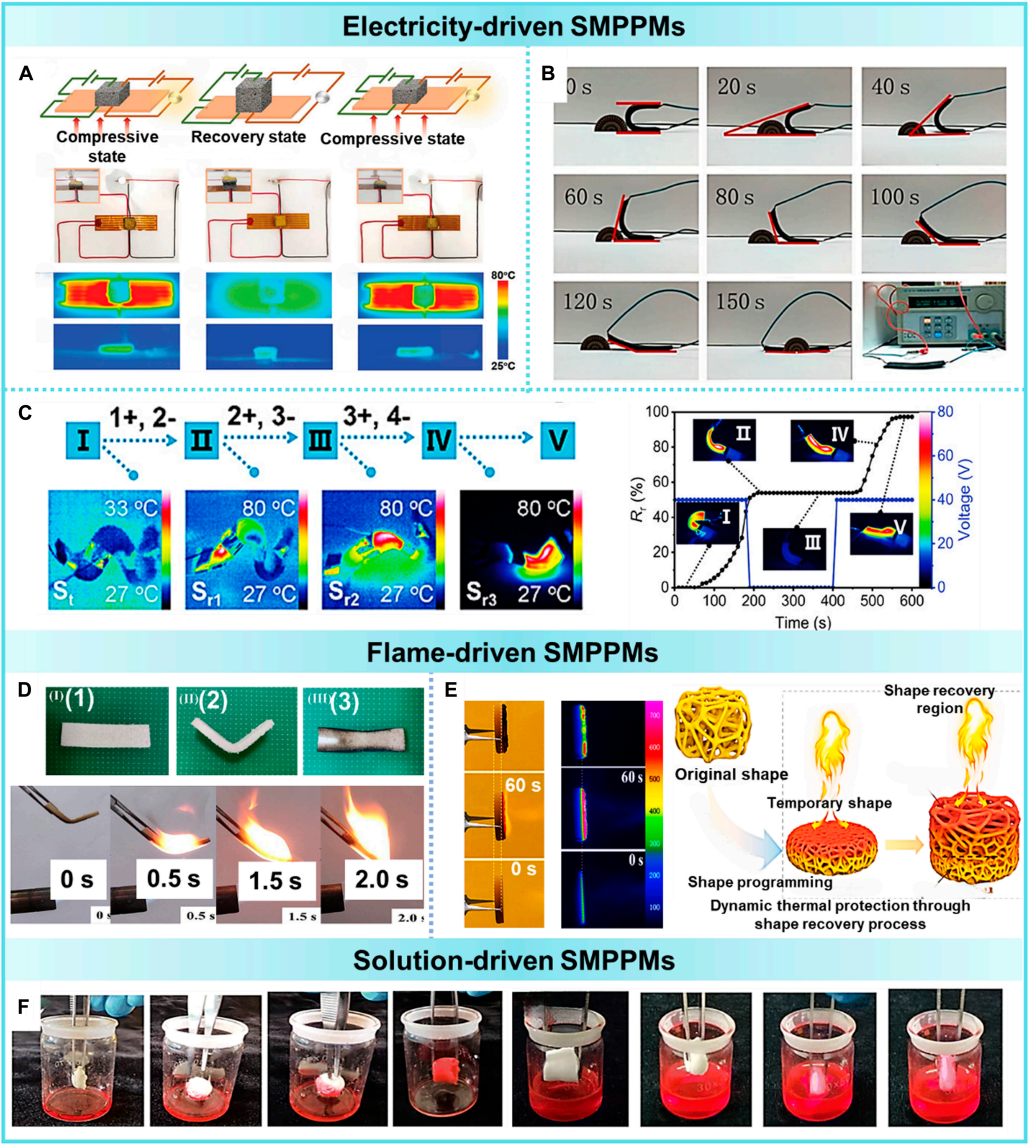
Other actuation methods of SMPPMs. (A to C) Electricity-driven shape recovery process of SMPPMs. Reprinted with permission from [23, 43, 103]. (D and E) Flamel-driven shape recovery process of SMPPMs. Reprinted with permission from [36, 106]. (F) Solution-driven shape recovery process of SMPPMs. Reprinted with permission from [107].

Zhao et al. [23] introduced liquid metal into the skeleton of SMEP foam, enabling the realization of conductive SMP foam composites. When the foam is compressed into a temporary shape, its porosity decreases, allowing the formation of an internal conductive path that facilitates shape recovery (Fig. 10A). Moreover, the resistance of the foam can be regulated by adjusting the compression ratio, enabling compatibility with various circuit requirements. Kausar et al. [103] fabricated a conductive SMPU composite foam by incorporating 8% graphene and 3% CNTs into the polyurethane matrix. The composite can complete shape recovery within 150 s under a voltage of 100 V (Fig. 10B). The multi-responsive SMPA composite developed by Huang et al. [43] can achieve electrically driven shape recovery within 190 s, with a shape recovery rate of 97.2% (Fig. 10C). Ding et al. [104] prepared multi-responsive shape memory foam composites by carbonizing polytriaminotriazine foam and subsequently compounding it with polycaprolactone. These composites are capable of shape recovery under both light and electrical stimulation, demonstrating potential applications in the field of sensing technologies.

### Other responsive

To overcome the limitations associated with single-stimulus actuation and broaden the application potential of SMPPMs, researchers have investigated alternative actuation methods [105]. The interaction between polar molecules and alternating magnetic fields in microwave environments enables rapid and uniform heating of the sample. As a relatively novel and efficient heating technique, microwave heating has gained increasing importance in chemical synthesis and material processing. Zhang et al. [54] fabricated shape memory polycaprolactone foams using microwave foaming technology and systematically compared the effects of microwave, oven, and water bath heating on the shape recovery behavior of SMPFs. The results demonstrated that microwave-driven SMPFs exhibited significantly faster shape recovery compared to other heating methods. SMPFs containing magnetic particles exhibit magnetically responsive actuation properties. However, the incorporation of magnetic particles typically alters the foam’s density, which may influence its mechanical characteristics.

Some researchers have further explored the shape recovery behavior of SMPPMs in extreme flame environments. The flame-driven mechanism depends on a material’s thermal response. To ensure operational integrity under direct flaming, such materials must possess paramount resistance to high temperatures and combustion. You et al. [106] developed a high-temperature resistant SMPI aerogel with inherent flame-retardant properties, which demonstrated complete shape recovery within 2 s when exposed to an open flame (Fig. 10D). Hu et al. [36] fabricated an ablation-resistant aerogel using shape memory phthalonitrile resin as the matrix material. This aerogel exhibited shape recovery under exposure to a 1,300 °C butane torch flame, as illustrated in Fig. 10E. Notably, during the recovery process, the aerogel increased its internal porosity, thereby enhancing its thermal insulation performance. This unique capability enables the regulation of thermal insulation through shape memory functionality, showing promising potential for applications in reusable aerospace vehicles. Certain SMPs possess a molecular structure rich in hydroxyl groups. These groups become distributed on the material’s surface following fabrication, contributing to its hydrophilic nature. Furthermore, the porous architecture of these SMPs offers a high specific surface area, which facilitates water- and solution-responsive behavior. Yu et al. [89] prepared water-responsive SMPAs with enhanced water absorption capacity by incorporating carboxyl-modified CNTs into PVA. These SMPAs achieved shape memory recovery through water activation following 75% compressive deformation. Hu et al. [35] reported water-driven shape memory behavior in a developed shape memory phenolic aerogel. Distinctively, this material requires hot water to trigger shape recovery, with the hot water-induced recovery time being 3 times faster than conventional heat-driven recovery. Cao et al. [107] reported a solvent-responsive and hydrophobic foam that exhibited rapid shape recovery in dichloromethane solution, while remaining inert in water, as shown in Fig. 10F.

## Application of SMPPMs

SMPPMs have achieved widespread applications in aerospace structures, biomedical devices, smart sensors, and soft robotics by virtue of their tunable porosity, stimulus-responsive behavior, light weight, and high compressibility. This section highlights representative research progress and technological developments, demonstrating the potential of SMPPMs to address specific engineering challenges and functional needs. Furthermore, through the integration of multifunctional capabilities such as self-sensing and intelligent actuation, the application scope of SMPPMs continues to extend into increasingly complex systems.

### Aerospace

**Fig. 11.**
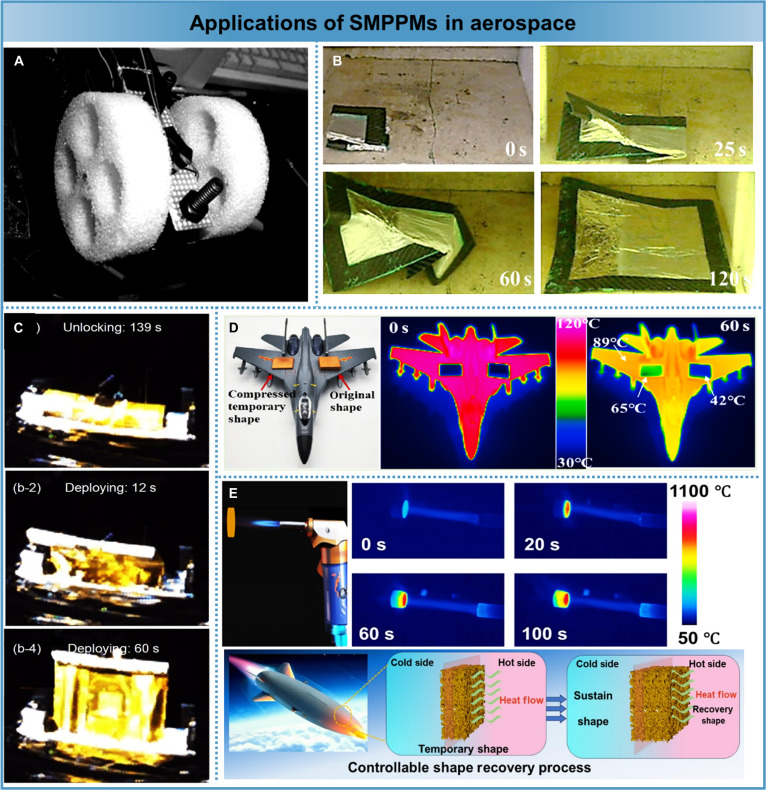
Aerospace applications of SMPPMs. (A) In actuators. Reprinted with permission from [85]. (B) In solar sails. Reprinted with permission from [109]. (C) In space deployable structure. Reprinted with permission from [110]. (D) Aircraft thermal protection systems. Reprinted with permission from [35]. (E) Infrared invisibility. Reprinted with permission from [36].

Conventional aerospace materials like magnesium–aluminum alloys, iron–nickel alloys, and high-temperature ceramics face challenges of high density and cost. SMPs and their composites have thus gained attention for aerospace applications due to advantages including compact compressibility, ease of transport/storage, and suitability for deployable space structures [108]. In 2003, JPL and MHI developed SMPF-based self-deploying structures for space load bearing. However, SMPFs’ structural limitations necessitated integration with other components. Addressing this, Composite Optics Inc. and JPL proposed a 2004 foam truss design with SMPF core and carbon fiber-reinforced SMP composite skins, suitable for large deplorables like space telescopes and solar arrays. CRG Corporation explored SMPF honeycombs in trans-medium drone wings to maintain airfoil profiles during transformation. Some researchers prepared SMEP foams via solid foaming and designed space actuators through ground tests, as shown in Fig. 11A [85]. Santo [109] further investigated the potential of SMPFs in solar sail deployment, proposing the incorporation of carbon nanotubes to enhance the stiffness of SMPFs. The solar sail could be compressed, packaged, and launched into space, where exposure to sunlight would trigger shape recovery and deployment of the sail. The shape memory behavior of this composite material is illustrated in Fig. 11B. Leng’s research group [110] achieved a pioneering in-orbit demonstration of an SMP-based flexible solar array system (SMPC-FSAS), validating SMP composites for ultra-large deployable space structures (Fig. 11C).

Aerospace reusable vehicle advancement demands flexible thermal protection systems. During cross-medium operations, varying heat flux environments require adaptive thermal strategies. Conventional ceramic aerogels occupy excessive structural space, making them unsuitable for compact designs. SMPAs offer deformable functionality: compressible into temporary configurations for minimized spatial occupancy, with thermal stimuli triggering shape recovery to enable effective insulation. Hu et al. [35] developed shape memory phenolic aerogels with exceptional thermal stability. When exposed to flames, the surface temperature difference can reach 600 °C, highlighting their thermal protection potential (Fig. 11D). They also elucidated SMPAs’ thermal insulation regulation mechanism through shape memory. Beyond thermal insulation, some SMPPMs enable tunable electromagnetic shielding via shape recovery. Jia et al. [111] designed a novel electromagnetic interference shielding material using compressible carbon foam repeatedly coated with TPI-MXene shape memory layers. Testing showed that CTAB (hexadecyl trimethyl ammonium bromide)-MXene loading up to 20 wt % enabled shielding effectiveness modulation through compressive strain. The material also exhibited excellent thermal/electrical stimulus-responsive shape memory for facile deformation/recovery. Hu et al. [36] also developed shape memory phthalonitrile aerogels with infrared stealth capabilities, indicating promising applications in aerospace platforms (Fig. 11E).

### Biomedicine

With the advancement of intelligent medical strategies, SMPPMs that exhibit intelligent responsiveness and excellent biocompatibility have demonstrated transformative potential in the biomedical field [112–114]. The design of their microstructure plays a crucial role in their performance in the biological environment. The micro- and nanoscale architecture, ultra-high compressive deformation capacity, and body-temperature-triggered shape memory behavior of SMPPMs make them ideal candidates for minimally invasive therapeutic applications [115–117]. Their nanoscale 3D framework and exceptionally large specific surface area enable efficient drug loading with minimal material usage, thereby enhancing drug delivery efficiency. Specifically, open-cell and interconnected porous networks are essential for high drug payloads and subsequent diffusion-based release profiles. The magnetic- and near-infrared light-responsive actuation mechanisms allow SMPPMs to achieve precise and controllable responses within complex biological environments. Recent studies have reported the successful application of SMPPMs in vascular embolization devices, tissue engineering, bone regeneration scaffolds, and hemostatic sponges, often leveraging specific structural designs for enhanced functionality.

**Fig. 12.**
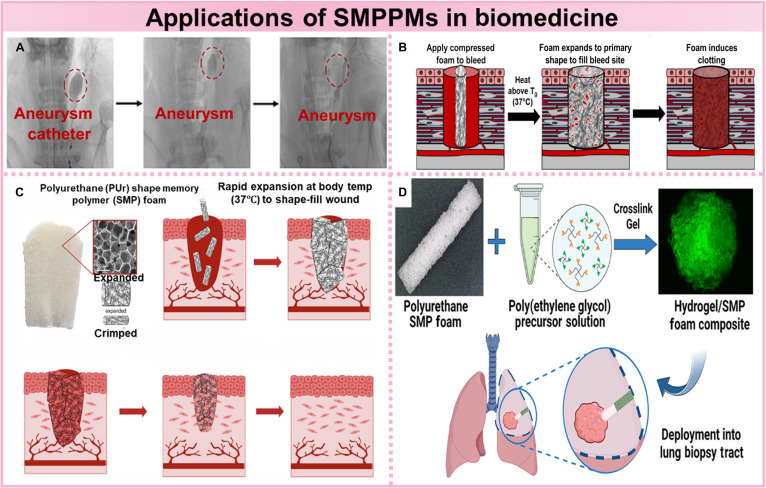
Biomedical applications of SMPPMs. (A) Aneurysm treatment. Reprinted with permission from [95]. (B and C) Hemostatic dressing. Reprinted with permission from [76, 118]. (D) Pulmonary biopsy channel sealant. Reprinted with permission from [63].

SMPPMs treat aneurysms by being compressed for catheter delivery and expanding upon thermal stimulus at the target site to occlude blood flow. By 2017, an embolization system based on SMPFs had received market approval, with the device being adaptable to various sizes for clinical use. Chen et al. [95] applied SMPFs in embolization sponge therapy for carotid artery sidewall aneurysms. In vitro embolization model experiments demonstrated that the sponge effectively isolated arterial blood flow from the aneurysm sac, as shown in Fig. 12A. The foam’s open-cell structure is crucial here, facilitating rapid blood to trigger expansion and ensuring a secure, conformal fit within the irregular aneurysm cavity. SMPPMs show applicability in wound hemostasis. SMPPMs show applicability in wound hemostasis. Christmas et al. [118] reported a blood-absorbing sponge capable of rapidly expanding to fill wound cavities and proposed a standardized application procedure for its use, as illustrated in Fig. 12B. This rapid expansion and filling efficacy is directly enabled by a highly porous, interconnected microstructure that maximizes capillary action and fluid uptake. Zhu et al. [119] developed SMPF hemostatic sponges demonstrating complete biodegradation within 14 d in mice, along with superior blood absorption, faster hemostasis, and reduced blood loss versus conventional materials. Petryk et al. [76] reported a hemostatic dressing based on SMPU (Fig. 12C), which can be compressed into a flat configuration for delivery to the wound site and subsequently recover its original shape upon contact with blood, simultaneously promoting coagulation. Vakil et al. [120] described an SMPU foam that enhances collagen deposition and supports cellular regeneration, making it suitable for implantation in wound sites to facilitate tissue repair and healing. For such tissue ingrowth applications, a gradient or bimodal pore structure is particularly advantageous, as larger pores facilitate cell migration and vascularization, while smaller pores enhance capillary force for hemostasis. Additionally, Jungmann et al. [63] developed PEG hydrogel–SMPF composites as lung biopsy sealants. Their shape recovery within tissue occludes biopsy channels, preventing blood/air leakage and reducing pneumothorax risk, as illustrated in Fig. 12D.

### Smart sensors

SMPPMs exhibit programmable response characteristics and strain–electrical signal coupling capabilities. When heated above their shape transition temperature, these materials demonstrate super-elastic behavior within their porous structures, enabling intelligent conversion of mechanical deformation into electrical signals. The exceptionally high porosity of SMPPMs imparts remarkable compression sensitivity, allowing for significant resistance changes under minimal external forces—performance that even surpasses that of traditional metal strain gauges. As a result, SMPPMs show considerable promise for use in sensor applications.

**Fig. 13.**
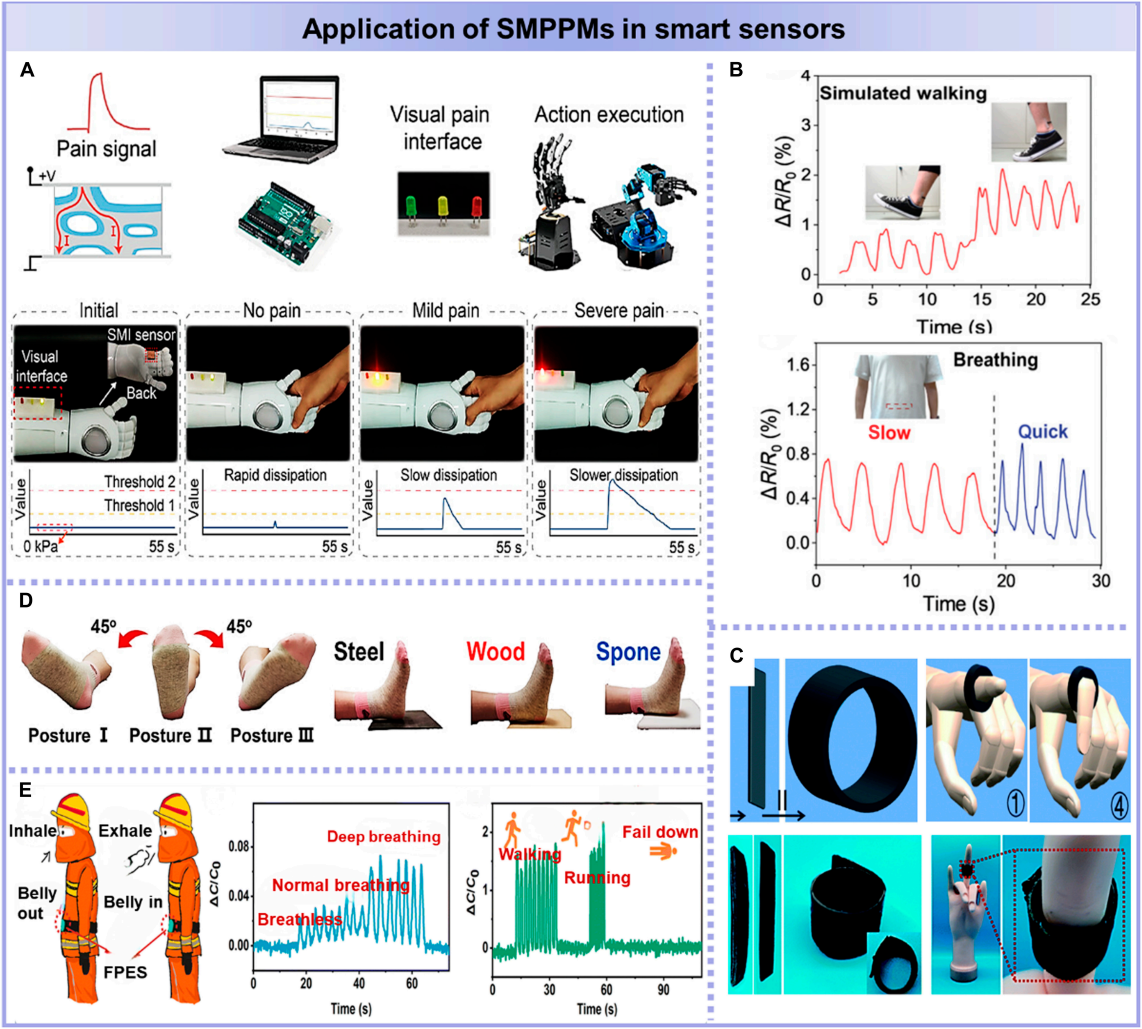
Application of SMPPMs in sensors. (A) Pain sensor. Reprinted with permission from [121]. (B) Motion sensor. Reprinted with permission from [122]. (C) Finger pressure sensor. Reprinted with permission from [44]. (D) Ankle pressure sensor. Reprinted with permission from [43]. (E) Symptom sensor. Reprinted with permission from [123].

Liu et al. [121] developed a biomimetic pain perception system based on SMPAs, capable of detecting and responding to varying levels of mechanical pain stimuli. The system consists of a pain signal collector, a signal processing unit, and a signal output module. The SMI (seamless integration of sensing and memory) sensor embedded in the pain signal collector can simulate and classify pain sensations based on applied pressure levels. Under different pressure conditions, the sensor distinguishes distinct pain intensities and transmits corresponding signals to the processor, which then activates specific color indicators on the output unit, as illustrated in Fig. 13A. Rong et al. [122] fabricated shape memory foam composites by integrating graphene foam with SMPs, thereby endowing the material with self-sensing capabilities during deformation. The developed SLGF (single-layer-graphene-dominated graphene foam) sensor utilizes the porous conductive network of graphene foam to convert mechanical strain into measurable electrical signals. The microscale porous structure undergoes deformation within a strain range of 0.03% to 37%, resulting in substantial resistance fluctuations (over 200% resistance change rate) due to variations in the contact resistance between graphene sheets. This enables the sensor to capture motion across multiple scales. When applied to the skin, the sensor can monitor physiological signals such as joint movements, respiratory rates, and gait patterns during running. Attachment to the throat allows recognition of speech patterns such as “hi” or “hello”, while placement on the neck enables the extraction of detailed pulse waveforms corresponding to a heart rate of 65 beats per minute. These functionalities demonstrate the advanced capabilities of intelligent sensing systems, as shown in Fig. 13B.

Huang et al. [44] developed a shape memory conductive aerogel [LSMC (lamellar shape-memory conductive)] capable of real-time detection of finger movements, aiming to prevent pressure-induced injuries. LSMC-based sensors can be fabricated into ring-shaped wearable devices for monitoring finger flexion, as shown in Fig. 13C. In a related study, Huang et al. [43] also applied LSMC sensors to monitor pressure distribution at the heel and ankle regions, enabling healthcare professionals to perform real-time patient status assessments, as depicted in Fig. 13D. Wang et al. [123] developed a flexible piezoelectric electronic skin (FPES) with sandwich architecture based on SMPPMs for fire rescue applications. The FPES detects elevated temperatures/mechanical impacts while enabling real-time physiological monitoring to enhance rescuer safety. A waistband-integrated sensor monitors respiration via capacitance fluctuations during inhalation/exhalation. FPES embedded in rescue footwear soles tracks motion, providing multi-parameter feedback on respiration, movement, and inactivity (Fig. 13E).

### Other applications

**Fig. 14.**
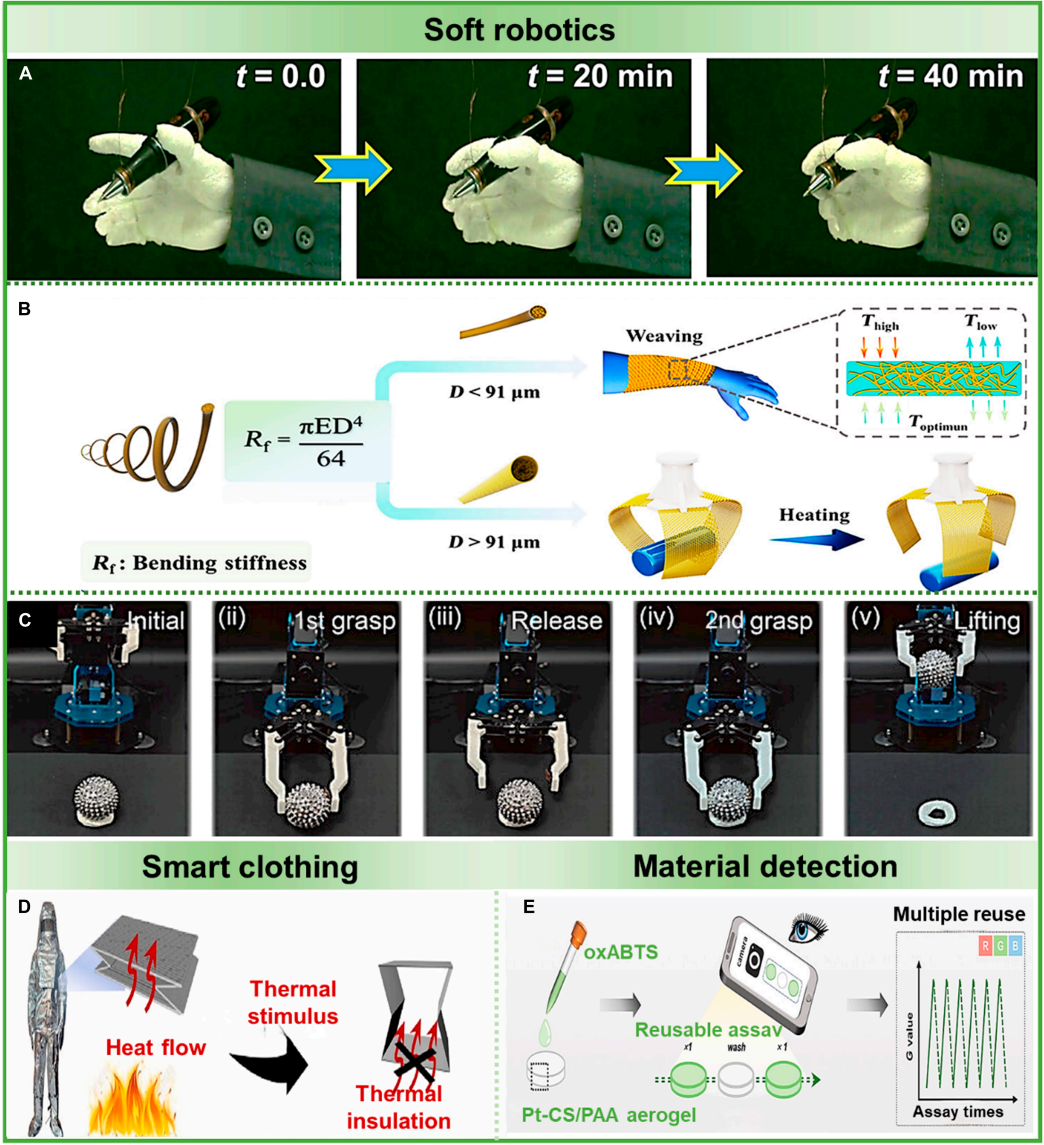
Other applications of SMPPMs. (A) Hand robot. Reprinted with permission from [78]. (B and C) Crawler robot. Reprinted with permission from [121, 126]. (D) Smart clothing. Reprinted with permission from [49]. (E) Material detection. Reprinted with permission from [61].

SMPPMs demonstrate broad applicability across diverse fields. In recent years, with the advancement of SMP research, SMPs and their composite materials have been increasingly applied in the field of soft robotics [124,125]. Porous-structured SMPs, in particular, demonstrate extensive application potential in this domain. SMPAs developed by Donthula et al. [78] can be fabricated into biomimetic hands capable of stably grasping a pen (Fig. 14A). Several SPM (smart porous materials)-based robotic systems are also capable of performing object grasping and releasing tasks. Bao et al. [126] designed a dynamic gripper using SMPPMs that can grasp objects at low temperatures and release them upon heating-induced softening. This gripper further incorporates photo-responsive functionality, enabling object release under infrared light or standard bulb illumination, as shown in Fig. 14B. Liu et al. [121] developed a gripper with integrated pain-sensing capability, enabling adaptive grasping of objects. When grasping irregular or spiky objects, the SPM-based sensor monitors contact pressure in real time. Once the preset “severe pain” threshold is exceeded, the system automatically triggers the release mechanism to discard the object. Subsequently, the gripper autonomously adjusts its grasping force and speed for subsequent attempts, ultimately achieving stable and safe grasping performance, as depicted in Fig. 14C.

Certain SMPPMs exhibit temperature-responsive behavior, making them suitable for use in intelligent textile systems. Shan et al. [62] fabricated ultra-flexible aerogel composite materials that maintain thermal insulation performance in both dry and submerged environments, making them ideal for personal thermal regulation in island or maritime settings. Fu et al. [49] reported printable SMPA composites with customizable molding capabilities, effectively addressing the processing challenges associated with traditional aerogel materials. The incorporation of a high-temperature resistant PI matrix endows these aerogels with promising applications in fire protection systems (Fig. 14D). Jia et al. [61] synthesized SMPPMs with peroxidase catalytic activity by embedding platinum-based nanoparticles with peroxidase-like activity into a polyacrylic acid aerogel matrix. This multifunctional material combines shape memory effects, nano-enzyme catalytic performance, and selective adsorption characteristics, achieving high sensitivity and repeatable detection of hydrogen peroxide (Fig. 14E). SMPPMs also find utility in automotive engineering.

## Conclusion and Prospect

SMPPMs represent a cutting-edge branch of smart materials, integrating the inherent advantages of porous structures (lightweight, high specific surface area, micro- and nanostructures) with the programmable deformation capabilities of SMPs, thereby enabling a chain-like development of structure–function–application integration. A comprehensive material system (including low-temperature, medium-temperature, and high-temperature systems) enables them to satisfy the demands of various application fields. Diverse preparation strategies (gas foaming, template method, 4D printing, freeze-drying, etc.) and design of micro/nanostructures allow for tailored performance and functional design. A variety of stimulus-driven mechanisms (thermal, optical, electrical, magnetic, and solvent-responsive) enable tunable, localized, and precise shape recovery. Through ingenious molecular design and structural regulation, SMPPMs not only achieve efficient and controllable shape memory effects but also endow the material with functions such as ultra-high porosity, ultra-low density, excellent thermal insulation/adsorption/catalytic performance, tunable electromagnetic shielding, biocompatibility, and multi-stimulus responsiveness, demonstrating immense application potential and revolutionary prospects in fields such as aerospace deployable structures and smart thermal protection, biomedicine, flexible sensing, biomimetic robotics, and smart textiles.

Despite significant progress in the research of SMPPMs, their future development still faces numerous challenges and opportunities. Future research should prioritize 4 directions. (a) Precise control of complex structures and synergistic optimization of performance are critical. Further investigation is needed into the relationship between multi-level pore structures (micro/nanometer pore synergy) and shape memory properties, mechanical strength, and functional characteristics (such as thermal conductivity/electrical conductivity and bioactivity). More refined preparation techniques (such as high-precision 4D printing) should be developed to achieve on-demand design of microstructures and programmable macrolevel performance. (b) Adapting to the trend toward diversified and intelligent actuation mechanisms is essential. New strategies for efficient, low-energy multi-field coupling actuation must be developed, along with improvements in response speed and environmental adaptability. (c) Overcoming the bottleneck in large-scale fabrication is critical. Current fabrication strategies for SMPPMs are primarily suited for laboratory-scale production of small samples. How to achieve large-scale fabrication of SMPPMs remains an area requiring further research. (d) Enhancing environmental sustainability of materials and manufacturing processes. There is a growing need to develop eco-friendly SMPPMs by using biodegradable or bio-based polymers and reducing the use of hazardous chemicals during synthesis and processing. Future efforts should also focus on green fabrication routes—such as solvent-free, energy-efficient, or low-emission techniques—to minimize environmental impact and improve the biocompatibility and recyclability of SMPPMs. (e) Continue to explore the deep expansion of application scenarios and interdisciplinary integration. We suggest conducting further exploration of the application of SMPPM in emerging fields such as soft robots, energy management of wearable intelligent devices, adaptive stealth, environmental restoration, and intelligent implantable devices, thereby promoting the interdisciplinary integration of materials science, mechanics, bionics, and artificial intelligence.

## References

[1] Yu Y, Zhang F, Liu Y, Leng J. Smart polymer fibers: Promising advances in microstructures, stimuli-responsive properties and applications. Adv Fiber Mater. 2025;7(4):1010–1041.

[2] Wang F, He W, Dai B, Zhang X, Wen Y. Recent advances in asymmetric wettability dressings for wound exudate management. Research. 2025;8:0591. 39810852 10.34133/research.0591 PMC11729271

[3] Jiang W, Liu C, Liu W, Zheng L. Advancements in intelligent sensing technologies for food safety detection. Research. 2025;8:0713. 40458611 10.34133/research.0713 PMC12128931

[4] Long Q, Jiang G, Zhou J, Zhao D, Yu H. A cellulose ionogel with rubber-like stretchability for low-grade heat harvesting. Research. 2024;7:0533. 39559347 10.34133/research.0533 PMC11570788

[5] Lu B, Hu E, Ding W, Wang W, Xie R, Yu K, Lu F, Lan G, Dai F. Bioinspired hemostatic strategy via pulse ejections for severe bleeding wounds. Research. 2023;6:0150. 37223487 10.34133/research.0150 PMC10202099

[6] Liu YK, Wang LL, Liu YJ, Zhang FH, Leng JS. Recent progress in shape memory polymer composites: Driving modes, forming technologies, and applications. Compos Commun. 2024;51: Article 102062.

[7] Kang D, Jeong J-M, Jeong KI, Kim SS. Improving the deformability and recovery moment of shape memory polymer composites for bending actuators: Multiple neutral axis skins and deployable core. ACS Appl Mater Interfaces. 2023;15(28):33944–33956. 37358080 10.1021/acsami.3c02590 PMC10360064

[8] Peng Q, Wang S, Han J, Huang C, Yu H, Li D, Qiu M, Cheng S, Wu C, Cai M. Thermal and magnetic dual-responsive catheter-assisted shape memory microrobots for multistage vascular embolization. Research. 2024;7:0339. 38550780 10.34133/research.0339 PMC10976590

[9] Liu Z, Lan X, Bian W, Liu L, Li Q, Liu Y, Leng J. Design, material properties and performances of a smart hinge based on shape memory polymer composites. Compos Part B Eng. 2020;195: Article 108056.

[10] Gao H, Li J, Liu Y, Leng J. Shape memory polymer solar cells with active deformation. Adv Compos Hybrid Mater. 2021;4(4):957–965.

[11] Ma JZ, Yang YZ, Valenzuela C, Zhang X, Wang L, Feng W. Mechanochromic, shape-programmable and self-healable cholesteric liquid crystal elastomers enabled by dynamic covalent boronic ester bonds. Angew Chem Int Ed Engl. 2022;61(9):202116219. 10.1002/anie.202116219 34962037

[12] Wang L, Zhang F, Liu Y, Leng J. Shape memory polymer fibers: Materials, structures, and applications. Adv Fiber Mater. 2022;4(1):5–23.

[13] Luo L, Zhang F, Wang L, Liu Y, Leng J. Recent advances in shape memory polymers: Multifunctional materials, multiscale structures, and applications. Adv Funct Mater. 2024;34(14):202312036.

[14] Asar A, Irfan MS, Khan KA, Zaki W, Umer R. Self-sensing shape memory polymer composites reinforced with functional textiles. Compos Sci Technol. 2022;221:109219.

[15] Jurinovs M, Veseta M, Sabalina A, Silva PES, Linarts A, Baniasadi H, Vapaavuori J, Gaidukovs S. Sustainable 4D printable biobased shape memory polymers with linear tunability and multistimuli actuation for advanced applications. Small Sci. 2025;5(7):2500104. 40666051 10.1002/smsc.202500104 PMC12257905

[16] Du WQ, Ren HP, Xu WL, Liu Y. Reversible shape memory behavior of knitting-fabric reinforced polymer matrix composites. Compos Commun. 2024;48: Article 101962.

[17] King O, Constant E, Weems AC. Shape memory poly(β-hydroxythioether) foams for oil remediation in aquatic environments. ACS Appl Mater Interfaces. 2021;13(17):20641–20652. 33872493 10.1021/acsami.1c02630

[18] Jang LK, Nash LD, Fletcher GK, Cheung T, Soewito A, Maitland DJ. Enhanced x-ray visibility of shape memory polymer foam using iodine motifs and tantalum microparticles. J Compos Sci. 2021;5(1):5010014.

[19] Xiao X, Panahi-Sarmad M, Xu R, Wang A, Cao S, Zhang K, Kamkar M, Noroozi M. Aerogels with shape memory ability: Are they practical—A mini-review. Eur Polym J. 2022;179: Article 111531.

[20] Shi W, Dong J, Liu J, Lu S. Progress in the structure and applications of smart phase change materials with shape memory properties. J Energy Storage. 2024;102: Article 114138.

[21] Dezaki ML, Bodaghi M. Soft magneto-responsive shape memory foam composite actuators. Macromol Mater Eng. 2022;307(11):2200490.

[22] Liang X, Sun Q, Zhang X, Hu Z, Liu M, Gu P, Yang X, Zu G. Advanced stretchable aerogels and foams for flexible electronics and beyond. Adv Funct Mater. 2024;34(48):2408707.

[23] Zhao R, Kang S, Wu C, Cheng Z, Xie Z, Liu Y, Zhang D. Designable electrical/thermal coordinated dual-regulation based on liquid metal shape memory polymer foam for smart switch. Adv Sci. 2023;10(8):2205428. 10.1002/advs.202205428 PMC10015848 36658714

[24] Elumalai D, Hosseinnezhad R, Bondarenko V, Morawiec J, Vozniak I, Galeski A. Shape memory polymer foam based on nanofibrillar composites of polylactide/polyamide. Molecules. 2024;29(21):29215045. 10.3390/molecules29215045 PMC11547612 39519686

[25] Du C, Fikhman DA, Persaud D, Monroe MBB. Dual burst and sustained release of p-coumaric acid from shape memory polymer foams for polymicrobial infection prevention in trauma-related hemorrhagic wounds. ACS Appl Mater Interfaces. 2023;15(20):24228–24243. 37186803 10.1021/acsami.3c04392 PMC10214375

[26] Du C, Fikhman DA, Obeng EE, Can SN, Dong KS, Leavitt ET, Saldanha LV, Hall M, Satalin J, Kollisch-Singule M, et al. Vanillic acid-based pro-coagulant hemostatic shape memory polymer foams with antimicrobial properties against drug-resistant bacteria. Acta Biomater. 2024;189:254–269. 39343289 10.1016/j.actbio.2024.09.036 PMC12090796

[27] Shi Z, Zhao G, Wang G, Zhang L, Wei C, Chai J. Development of ultralight, tough and hydrophobic polymethylmethacrylate/polyvinylidene fluoride shape memory foams for heat insulation applications. Mater Des. 2023;225: Article 111527.

[28] Chai J, Wang G, Zhao J, Zhang A, Shi Z, Wei C, Zhao G. Microcellular PLA/PMMA foam fabricated by CO_2_ foaming with outstanding shape-memory performance. J CO₂ Util. 2021;49: Article 101553.

[29] Walter M, Friess F, Krus M, Zolanvari SMH, Grun G, Krober H, Pretsch T. Shape memory polymer foam with programmable apertures. Polymers. 2020;12(9):12091914. 10.3390/polym12091914 PMC7565147 32854329

[30] Ren X, Wang J, Wang T, Li Z, Han S, Sun G. Fabrication of one-step shape memory gradient sound absorber with wrinkled inner wall and closed-pore structure. Eur Polym J. 2023;196: Article 112226.

[31] Tian Y, Feng X, Wang C, Shang S, Liu H, Huang X, Jiang J, Song Z, Zhang H. Fully biobased degradable vitrimer foams: Mechanical robust, catalyst-free self-healing, and shape memory properties. ACS Appl Mater Interfaces. 2024;16(5):6523–6532. 38275160 10.1021/acsami.4c00267

[32] Zhou Y, Liu W, Zhang S, Liu H, Wu Z, Wang X. Eco-friendly flame-retardant phase-change composite films based on polyphosphazene/phosphorene hybrid foam and paraffin wax for light/heat-dual-actuated shape memory. ACS Appl Mater Interfaces. 2024;16(6):7754–7767. 38306229 10.1021/acsami.3c16953

[33] Hui J, Xu F, Ni Q-Q. Temperature-stimulated phase switching in a novel reversible morphing shape memory sponge for soft robotic actuators. Chem Eng J. 2025;505: Article 159177.

[34] Chen J, Jiang W-J, Zeng Z, Sun D-X, Qi X-D, Yang J-H, Wang Y. Multifunctional shape memory foam composites integrated with tunable electromagnetic interference shielding and sensing. Chem Eng J. 2023;466: Article 143373.

[35] Hu L, Luo L, Zhang F, Liu Y, Leng J. Self-sensing shape memory boron phenolic-formaldehyde aerogels with tunable heat insulation for smart thermal protection systems. Chem Eng J. 2025;505: Article 159558.

[36] Hu R, Zhang F, Luo L, Wang L, Liu Y, Leng J. Reconfigurable high-temperature thermal protection shape memory aerogel based on phthalonitrile resin with facile template method. Carbon. 2025;242: Article 120378.

[37] Zhao L, Wang L, Shi J, Hou X, Wang Q, Zhang Y, Wang Y, Bai N, Yang J, Zhang J, et al. Shape-programmable interfacial solar evaporator with salt-precipitation monitoring function. ACS Nano. 2021;15(3):5752–5761. 33683874 10.1021/acsnano.1c01294

[38] He Y-J, Shao Y-W, Xiao Y-Y, Yang J-H, Qi X-D, Wang Y. Multifunctional phase change composites based on elastic MXene/silver nanowire sponges for excellent thermal/solar/electric energy storage, shape memory, and adjustable electromagnetic interference shielding functions. ACS Appl Mater Interfaces. 2022;14(4):6057–6070. 35042328 10.1021/acsami.1c23303

[39] Wang H-Y, Liu D, Zhang K, Qian C, Dong Y, Jamaluddin N, Matmin J, Zhu Y. Shape memory HCNTs/PANI/WPU aerogels as dynamically tunable microwave absorbers in response to mechanical deformation. J Alloys Compd. 2025;1036: Article 181930.

[40] Zhou D, Xue T, Fu Z, Wang S, Zhang X, Fan W, Liu T. Lightweight fluorinated block copolyimide aerogels for high-temperature shape memory. Polymer. 2025;328: Article 128430.

[41] Guo M, Zhang Y, Huang C, Zhao X, Yan X-P, Huang Y, Li L, Liu T. Shape memory polyimide aerogel composites with high programming temperatures and exceptional shape recovery capability. Compos Part A Appl Sci Manuf. 2023;174: Article 107717.

[42] Li B, Xiao Y, Du L, Fan C-J, Xiao W-X, Yang K-K, Wang Y-Z. Fabrication of shape-memory aerogel based on chitosan/poly (ethylene glycol) diacrylate semi-interpenetrating networks via a facile and eco-friendly strategy. Macromol Mater Eng. 2019;304(8):201900169.

[43] Huang J, Wang Y, Guo J, Wu S, Xie H, Zhou S. Anisotropic conductive shape-memory aerogels as adaptive reprogrammable wearable electronics for accurate long-term pressure sensing. J Mater Chem A. 2022;10(8):3933–3943.

[44] Huang J, Liu H, Chen Q, Xie H, Zhou S. Shape-programmable lamellar aerogel enabling 3D wireless point-of-care electronics for assistance in pressure injury prevention. Adv Funct Mater. 2025;35(13):202418037.

[45] Qi S, Tian X, Yuan W. High-stretchable solid-solid phase change aerogel surface-modified with Ni-MOF for efficient photothermal energy conversion-storage and thermal-induced shape programming. Adv Funct Mater. 2025.

[46] Momeni F, Ni J. Laws of 4D printing. Engineering. 2020;6(9):1035–1055.

[47] Guan Z, Wang L, Bae J. Advances in 4D printing of liquid crystalline elastomers: Materials, techniques, and applications. Mater Horiz. 2022;9(7):1825–1849. 35504034 10.1039/d2mh00232a

[48] Geng M, Liu T, Zhang X, Xue T, Fan W, Liu T. 4D printed shape memory polyimide composite aerogels with high recovery stress for load driving. ACS Appl Mater Interfaces. 2025;17(9):14615–14622. 39969217 10.1021/acsami.4c23086

[49] Fu Z, Yu D, Xue T, Zhang X, Fan W. 3D printed polyimide-based composite aerogels with shape memory and thermal insulation properties. Compos Commun. 2025;56: Article 102335.

[50] Fan D, Li N, Li Y, Xing H, Wang S, Li S, Jiang Z, Li M, Tang T. A novel method for preparing anisotropic negative Poisson’s ratio composite foam with excellent structural stability and shape recovery property. Polymer. 2024;296: Article 126825.

[51] Zhao G, Wang HM, You JA, Xing HP, Xue J, Jiang ZW, Tang T. Superlight flame-retardant poly(aryl ether ketone)/polyurea nanocomposite foam with excellent solvent-resistance and shape memory performance. Compos Commun. 2024;48: Article 101946.

[52] You J, Jiang Z, Jiang H, Qiu J, Li M, Xing H, Xue J, Tang T. A plasticizing-foaming-reinforcing approach for creating thermally insulating PVC/polyurea blend foams with shape memory function. Chem Eng J. 2022;450: Article 138071.

[53] Tian M, You J, Qiu J, Li M, Xing H, Xue J, Jiang Z, Tang T. Unexpected super anti-compressive styrene-acrylonitrile copolymer/polyurea nanocomposite foam with excellent solvent resistance, re-processability and shape memory performance. Compos Part B Eng. 2023;264: Article 110908.

[54] Zhang F, Zhou T, Liu Y, Leng J. Microwave synthesis and actuation of shape memory polycaprolactone foams with high speed. Sci Rep. 2015;5:srep11152. 10.1038/srep11152 PMC4459203 26053586

[55] Xiang P, Zhang H, Zhang Q, Yu Z, Wang Y, Li J, Lei J. Mild fabrication of polymeric porous/nonporous micro-composite structures via enhancing the photothermal conversion of ultrafast laser. Adv Funct Mater. 2024;34(51):10304.

[56] Pabst W, Uhlirova T, Gregorova E, Wiegmann A. Young’s modulus and thermal conductivity of closed-cell, open-cell and inverse ceramic foams model-based predictions, cross-property predictions and numerical calculations. J Eur Ceram Soc. 2018;38(6):2570–2578.

[57] Tarkashvand A, Daneshjou K, Golmohammadi A. FG and viscoelastic models combination for vibroacoustic modeling of sandwich structures made of open and closed cell foam materials. Compos Struct. 2021;259: Article 113438.

[58] Kim S, Kim DG, Kim M, Kim KJ, Lee JM, Lee JH, Cheong H-W, Kim HS, Lee S. Analyses of impact energy-absorbing performance of open- and closed-cell Al foams using modified split Hopkinson pressure bar. J Alloys Compd. 2023;965: Article 171349.

[59] Zhao W, Zeng C, Liu L, Leng J, Liu Y. Shape memory sandwich structure with reprogrammable shape and mechanical properties. Compos Struct. 2025;351: Article 118604.

[60] Tian Y, Dai S, Wang J, Huang X, Zhang H, Chen Y. Degradable and programmable shape-color dual-response tung oil-based foams with dynamic covalent bonds. Ind Crop Prod. 2024;222: Article 120121.

[61] Jia W, Zhu B, Zhang A, Hou H, Qu Z, Bu Y, Liu L, Du B. Hydrophilic shape-memory nanozyme aerogel for the development of a reusable and signal-amplified sensor. Adv Funct Mater. 2025;35(23):18910.

[62] Shan X, Hu P, Wang J, Liu L, Yuan D, Zhang J, Wang J. Super-stretchable hybrid aerogels by self-templating strategy for cross-media thermal management. Macromol Rapid Commun. 2023;44(8):2200948. 10.1002/marc.202200948 36700486

[63] Jungmann MA, Phillips SR, Touchet TJ, Brinson B, Parish K, Petersen C, Hasan SM, Nash LD, Maitland DJ, Alge DL. Swellable and thermally responsive hydrogel/shape memory polymer foam composites for sealing lung biopsy tracts. ACS Biomater Sci Eng. 2023;9(2):642–650. 36729490 10.1021/acsbiomaterials.2c01369 PMC10726527

[64] Hu L, Zhang F, Luo L, Wang L, Liu Y, Leng J. Design and preparation of shape memory phenol-formaldehyde foam composites with excellent thermal stability and mechanical properties. Compos Part A Appl Sci Manuf. 2023;174: Article 107738.

[65] Studart AR, Gonzenbach UT, Tervoort E, Gauckler LJ. Processing routes to macroporous ceramics: A review. J Am Ceram Soc. 2006;89(6):1771–1789.

[66] Luo L, Zhang F, Pan W, Yao Y, Liu Y, Leng J. Shape memory polymer foam: Active deformation, simulation and validation of space environment. Smart Mater Struct. 2022;31(3):035008.

[67] Zhang Y, Wang X, Guo M, Li L, Liu T. Homogenous nanofiber skeleton-reinforced shape memory polyimide aerogel composites with high deformability and rapid recovery. Compos Part A Appl Sci Manuf. 2025;194: Article 108907.

[68] Liu Z, He F, Yang A, Su L, Li Y, Jiang S, Chen Z, Yang W. Double-skeleton based shape-stabilized phase change materials with excellent solar-thermal energy conversion and shape memory performance. Thermochim Acta. 2022;717:179360.

[69] Li SW, Hou DM, Cui YS, Jia S, Lan G, Sun WL, Li GY, Li X, Feng W. Highly ordered carbon aerogels: Synthesis, structures, properties and applications. Carbon. 2024;218: Article 118669.

[70] Zhang L, Li X, Ding E, Guo Z, Luo C, Zhang H, Yu J. Shape memory polyimide/carbon nanotube composite aerogels with physical and chemical crosslinking architectures for thermal insulating applications. Compos Sci Technol. 2024;251: Article 110588.

[71] Liu Y, Zhang Y, Xiong X, Ge P, Wu J, Sun J, Wang J, Zhuo Q, Qin C, Dai L. Strategies for preparing continuous ultraflexible and ultrastrong poly(vinyl alcohol) aerogel fibers with excellent thermal insulation. Macromol Mater Eng. 2021;306(11):2100399.

[72] Li C, Wang X, Zhang C, Zhang Z, Zhou B, Shen J. Elastic, flexible, and tough hierarchical polyimide aerogels with dynamic reversible physical entanglement structure. Chem Eng J. 2025;509: Article 161429.

[73] Hargett SE, Lokhande GK, Duran J, Hirani Z, Jang LK, Foster S, Deo KA, George S, Javed M, Ware TH, et al. Nanoengineered shape-memory hemostat. Small Sci. 2025;5(2):2400321. 40213067 10.1002/smsc.202400321 PMC11934902

[74] Kim HM, Park J, Huang ZM, Youn JR, Song YS. Carbon nanotubes embedded shape memory polyurethane foams. Macromol Res. 2019;27(9):919–925.

[75] Zhu S, Zhou Q, Wang M, Dale J, Qiang Z, Fan Y, Zhu M, Ye C. Modulating electromagnetic interference shielding performance of ultra-lightweight composite foams through shape memory function. Compos Part B Eng. 2021;204: Article 108497.

[76] Petryk NM, Thai NLB, Saldanha LV, Sutherland ST, Monroe MBB. Bioactive polyurethane shape memory polymer foam dressings with enhanced blood and cell interactions for improved wound healing. ACS Appl Mater Interfaces. 2025;17(18):26402–26415. 40261803 10.1021/acsami.5c02532 PMC12067373

[77] Liu P, Lai H, Luo X, Xia Q, Zhang D, Cheng Z, Liu Y, Jiang L. Superlyophilic shape memory porous sponge for smart liquid permeation. ACS Nano. 2020;14(10):14047–14056. 32970408 10.1021/acsnano.0c06673

[78] Donthula S, Mandal C, Leventis T, Schisler J, Saeed AM, Sotiriou-Leventis C, Leventis N. Shape memory Superelastic poly(isocyanurate-urethane) aerogels (PIR-PUR) for deployable panels and biomimetic applications. Chem Mater. 2017;29(10):4461–4477.

[79] Malakooti S, Doulah ABMSU, Ren Y, Kulkarni VN, Soni RU, Edlabadkar VA, Zhang R, Vivod SL, Sotiriou-Leventis C, Leventis N, et al. Meta-aerogels: Auxetic shape-memory polyurethane aerogels. ACS Appl Polym Mater. 2021;3(11):5727–5738.

[80] Doulah ABMSU, Mandal C, Far HM, Edlabadkar VA, Soni RU, Owusu SY, Leventis N, Sotiriou-Leventis C. Using catalysis to control the morphology and stiffness of shape memory poly(isocyanurate-urethane) (PIR-PUR) aerogels. ACS Appl Polym Mater. 2023;5(9):6851–6863.

[81] Wang C, Wang M, Ying S, Gu J. Fast chemo-responsive shape memory of stretchable polymer nanocomposite aerogels fabricated by one-step method. Macromol Mater Eng. 2020;305(1):1900602.

[82] Sauter T, Kratz K, Madbouly S, Klein F, Heuchel M, Lendlein A. Anisotropy effects in the shape-memory performance of polymer foams. Macromol Mater Eng. 2021;306(4):2000730.

[83] Ma C-Q, Shi W-Z, Liu J-S, Xing J-W, Li S-S, Huang Y-Y. Simultaneous phase change energy storage and thermoresponsive shape memory properties of porous poly(vinyl alcohol)/phase change microcapsule composites. Polym Int. 2021;70(6):803–811.

[84] Yan S, Zhang F, Luo L, Wang L, Liu Y, Leng J. Shape memory polymer composites: 4D printing, smart structures, and applications. Research. 2023;6:0234. 37941913 10.34133/research.0234 PMC10629366

[85] Luo L, Zhang F, Leng J. Shape memory epoxy resin and its composites: From materials to applications. Research. 2022;2022: Article 9767830. 35360647 10.34133/2022/9767830 PMC8949802

[86] Li L, Yang G, Lyu J, Sheng Z, Ma F, Zhang X. Folk arts-inspired twice-coagulated configuration-editable tough aerogels enabled by transformable gel precursors. Nat Commun. 2023;14(1):8450. 38114508 10.1038/s41467-023-44156-4 PMC10730912

[87] Wang W, Wang W, Liang Y, Du L, Yang H, Ma H, Cheng H, Yan Y, Shen Y, Chen Q. Advanced stimuli-responsive structure based on 4D aerogel and covalent organic frameworks composite for rapid reduction in tetracycline pollution. Molecules. 2023;28(14):5505. 37513377 10.3390/molecules28145505 PMC10386521

[88] Mohsenian Z, Kokabi M, Alamdarnejad G. Shape memory behaviour of polyvinyl alcohol aerogels cross-linked with Fe^3+^& Cu^2+^ metallic ions. Smart Mater Struct. 2023;32(8):085005.

[89] Yu C, Shi Q, Zhao H, Guo J, Lin D, Yao Y, Zhang X, Jiang X. Multilayer polyvinyl alcohol/carbon composite aerogels with broadband microwave and noise absorption and novel shape memory effect. Adv Funct Mater. 2025;35(43):02749.

[90] Liu T, Liu Y, Zhang Z, Xi S, Yu C, Zhang X, Yang J, Wang X, Zhang Z, Shen J. Ultra-light, elastic, magnetic polyimide/Fe_3_O_4_ composite aerogel with magnetic actuation function. J Porous Mater. 2022;29(6):1779–1789.

[91] Wang Y, Zhou DZ, Tang ZY, Xia Y, Lv Y, Wang ZC, Ma BM, Zhang X, Fan W, Liu TX. Highly loaded actuation achieved by shape memory block copolyimide aerogels with tunable distribution of stationary and reversible phases. Macromolecules. 2025;58(11):5852–5861.

[92] Li X, Guo Z, Yang P, Zhao B, Li J, Yin M, Liu W, Luo C, Zhang L. Regulating porous microstructure of polyimide aerogels toward efficient shape memory performance. Polymer. 2024;307:127224.

[93] Xia L, Ma Y, Wang Q, Meng J, Geng J. Facile fabrication of bio-based Eucommia Ulmoides rubber shape memory foams. Polym Test. 2022;106: Article 107455.

[94] Wang H, You J, Tian M, Qiu J, Xing H, Xue J, Jiang Z, Tang T. Preparing flame-retardant poly(phenylene oxide)/polyurea nanocomposite foam with excellent heat-resistance and shape memory performance. Compos Commun. 2023;40: Article 101589.

[95] Chen X, Zhang N, Ni C, Cao R, Hu L, Chen J, Zhao Q, Xie T, Liu Z. Geometrically adaptive porous shape memory polymers towards personalized biomedical devices. Chem Eng J. 2024;484: Article 149394.

[96] Wang T, Zhao J, Weng C, Wang T, Liu Y, Han Z, Zhang Z. Three-dimensional graphene coated shape memory polyurethane foam with fast responsive performance. J Mater Chem C. 2021;9(23):7444–7451.

[97] Wang M, Song Y, Bisoyi HK, Yang J-F, Liu L, Yang H, Li Q. A liquid crystal elastomer-based unprecedented two-way shape-memory aerogel. Adv Sci. 2021;8(22):2102674. 10.1002/advs.202102674 PMC8596101 34569166

[98] Wang M, Li J, Yang H. Dynamic diselenide bond-enabled liquid crystal elastomer-based two-way shape memory aerogels with weldability and closed-loop recyclability. Smart Mol. 2023;1(3): Article 230009. 10.1002/smo.20230009 PMC12118233 40626211

[99] Shi T, Liu H, Wang X. Multi-stimuli-responsive shape memory flexible composites based on magnetic melamine/polydopamine/phosphorene complex foams and polyethylene glycol. Compos Part A Appl Sci Manuf. 2024;181: Article 108117.

[100] Hu W-W, Shi X-Y, Gao M-H, Huang C-H, Huang T, Zhang N, Yang J-H, Qi X-D, Wang Y. Light-actuated shape memory and self-healing phase change composites supported by MXene/waterborne polyurethane aerogel for superior solar-thermal energy storage. Compos Commun. 2021;28: Article 100980.

[101] Wu H-Y, Li S-T, Shao Y-W, Jin X-Z, Qi X-D, Yang J-H, Zhou Z-W, Wang Y. Melamine foam/reduced graphene oxide supported form-stable phase change materials with simultaneous shape memory property and light-to-thermal energy storage capability. Chem Eng J. 2020;379: Article 122373.

[102] Chen C, Yu HT, Lai T, Guo J, Qin MM, Qu ZG, Feng YY, Feng W. Flexible and elastic thermal regulator for multimode intelligent temperature control. SusMat. 2023;3(6):843–858.

[103] Kausar A, Ahmad I, Zhao T, Aldaghri O, Ibnaouf KH, Eisa MH. Nanocomposite foams of polyurethane with carbon nanoparticles-design and competence towards shape memory, electromagnetic interference (EMI) shielding, and biomedical fields. Crystals. 2023;13(8):cryst13081189.

[104] Ding X, Shi Y, Xu S, Zhang Y, Du J, Qiu J. Triple stimuli-responsive flexible shape memory foams with super-amphiphilicity. Small. 2023;19(6):2205797. 10.1002/smll.202205797 36461700

[105] Zou YL, Guo WQ, Lu XL, Sun ZJ, Li L. A novel water-induced two-way shape memory polymer based on poly (L-lactic acid)/silk fibroin composites. Compos Commun. 2024;46: Article 101822.

[106] You J, Cai L, Yu R, Xing H, Xue J, Li Y, Jiang Z, Cui D, Tang T. High-performance chlorinated polyvinyl chloride/polyurea nanocomposite foam with excellent solvent resistance, flame-triggered shape memory effect and its upcycling. Compos Part A Appl Sci Manuf. 2024;177:107931.

[107] Cao J, Gui C, Feng S. Porous recyclable sponges with controllable and durable shape memory. Mater Adv. 2023;4(4):1075–1080.

[108] Santo L, Quadrini F, Bellisario D, Iorio L, Proietti A, de Groh KK. Effect of the LEO space environment on the functional performances of shape memory polymer composites. Compos Commun. 2024;48: Article 101913.

[109] Santo L. Shape memory composite structures for self-deployable solar sails. Astrodynamics. 2022;6(4):441.

[110] Lan X, Liu L, Zhang F, Liu Z, Wang L, Li Q, Peng F, Hao S, Dai W, Wan X, et al. World’s first spaceflight on-orbit demonstration of a flexible solar array system based on shape memory polymer composites. Sci China Technol Sci. 2020;63(8):1436–1451.

[111] Jia X, Shen B, Zhang L, Zheng W. Construction of shape-memory carbon foam composites for adjustable EMI shielding under self-fixable mechanical deformation. Chem Eng J. 2021;405:126927.

[112] Hicks AJ, Roberts C, Robinson A, Wilson K, Kotamreddy V, LaRue T, Veyssi A, Beltran F, Hakim J, Rausch MK, et al. Polycaprolactone-based shape memory foams as self-fitting vaginal stents. Acta Biomater. 2024;187:172–182. 39214160 10.1016/j.actbio.2024.08.041 PMC11600519

[113] Graul LM, Horn SJ, Nash LD, Cheung TB, Clubb FJ, Maitland DJ. Image-based evaluation of in vivo degradation for shape-memory polymer polyurethane foam. Polymers. 2022;14(19):14194122. 10.3390/polym14194122 PMC9571375 36236069

[114] Du C, Fikhman DA, Monroe MBB. Shape memory polymer foams with phenolic acid-based antioxidant properties. Antioxidants. 2022;11(6):1105. 35740002 10.3390/antiox11061105 PMC9219628

[115] Pineda-Castillo SA, Luo J, Lee H, Bohnstedt BN, Liu Y, Lee C-H. Effects of carbon nanotube infiltration on a shape memory polymer-based device for brain aneurysm therapeutics: Design and characterization of a joule-heating triggering mechanism. Adv Eng Mater. 2021;23(6):2100322.

[116] Monroe M, Beaman H, Vakil A, Du C. Shape memory polymer hydrogel foams for fistula closure. Gastroenterology. 2021;160(3):S22.

[117] Vakil AU, Petryk NM, Shepherd E, Monroe MBB. Biostable shape memory polymer foams for smart biomaterial applications. Polymers. 2021;13(23):13234084. 10.3390/polym13234084 PMC8658902 34883587

[118] Christmas N, Vakil AU, Hatch CJ, Dong S, Fikhman D, Beaman HT, Monroe MBB. Characterization of shape memory polymer foam hemostats in in vitro hemorrhagic wound models. J Biomed Mater Res Part B Appl Biomater. 2021;109(5):681–692. 10.1002/jbm.b.34732 32969163

[119] Zhu Y, Zhang L, Duan W, Martin-Saldana S, Li C, Yu H, Feng L, Zhang X, Du B, Li G, et al. Succinic ester-based shape memory gelatin sponge for noncompressible hemorrhage without hindering tissue regeneration. Adv Healthc Mater. 2023;12(5):2202122. 10.1002/adhm.202202122 36399015

[120] Vakil AU, Petryk NM, Du C, Howes B, Stinfort D, Serinelli S, Gitto L, Ramezani M, Beaman HT, Monroe MBB. In vitro and in vivo degradation correlations for polyurethane foams with tunable degradation rates. J Biomed Mater Res A. 2023;111(5):580–595. 36752708 10.1002/jbm.a.37504

[121] Liu L, Yu S, Xu Y, Chen H, Wang H, Lin W, Hu Y, Huang Z, Wei C, Lin Y, et al. Dynamically reversible filament networks enabling programmable in-sensor memory for high-precision neuromorphic interactions. Adv Funct Mater. 2025;35(34): Article 2504456.

[122] Rong J, Zhou J, Zhou Y, Hu C, Li L, Guo W. 3D single-layer-dominated graphene foam for high-resolution strain sensing and self-monitoring shape memory composite. Small. 2022;18(51):2205301. 10.1002/smll.202205301 36319465

[123] Wang W, Wang S, Li Z, Zhou J, Liu S, Xuan T, Zhang J, Gong X. A flexible protective electronic skin with tunable anti-impact and thermal insulation properties for potential rescue applications. J Mater Chem C. 2025;13(3): Article 175582.

[124] Khosroshahi Z, Karimzadeh F, Enayati MH, Kalali EN, Adabavazeh Z, Wallrabe U. Shape memory behavior of polyethylene-foam-based nanocomposite for sustainable triboelectric nanogenerators. J Alloys Compd. 2024;1003: Article 175582.

[125] Sarrafan S, Feng X, Li G. A soft syntactic foam actuator with high recovery stress, actuation strain, and energy output. Mater Today Commun. 2022;31: Article 103303.

[126] Bao Y, Lyu J, Liu Z, Ding Y, Zhang X. Bending stiffness-directed fabricating of kevlar aerogel-confined organic phase-change fibers. ACS Nano. 2021;15(9):15180–15190. 34423639 10.1021/acsnano.1c05693

